# Severity of All-Terrain Vehicle–Related Injuries by Age in Canada, 2002-2019

**DOI:** 10.1001/jamanetworkopen.2023.16060

**Published:** 2023-05-31

**Authors:** William MacDougall, Xuejing Jiang, Shamsia Sobhan, Robert Balshaw, Barbara Haas, Lynne Moore, Natalie Yanchar, Jonathan McGavock

**Affiliations:** 1 Children’s Hospital Research Institute of Manitoba, Winnipeg, Manitoba, Canada; 2 Department of Pediatrics, Rady Faculty of Health Sciences, University of Manitoba, Winnipeg, Manitoba, Canada; 3 George and Fay Yee Centre for Healthcare Innovation University of Manitoba, Winnipeg, Manitoba, Canada; 4 Department of Community Health Sciences, Rady Faculty of Health Sciences, University of Manitoba, Winnipeg, Manitoba, Canada; 5 Evaluative Clinical Sciences, Trauma, Emergency & Critical Care Program, Sunnybrook Research Institute, Toronto, Ontario, Canada; 6 Department of Social and Preventive Medicine, Universite de Laval, Quebec City, Quebec, Canada; 7 Alberta Children’s Hospital Research Institute, Calgary, Alberta, Canada; 8 Department of Surgery, University of Calgary, Calgary, Alberta, Canada

## Abstract

**Importance:**

Rates of pediatric hospitalizations following an injury while riding an all-terrain vehicle (ATV) are increasing. Few studies have compared the rates of severe injuries among children, adolescents, and adults.

**Objectives:**

To investigate whether younger ATV riders are at risk for more severe ATV-related injuries.

**Design, Setting, and Participants:**

This cross-sectional study was conducted using administrative data from hospitals in 9 provinces in Canada. Participants included patients admitted to hospitals with ATV-related injuries between 2002 and 2019. Statistical analysis was performed from June 2020 to September 2021.

**Exposures:**

The primary exposure was age younger than 16 years. Comparison groups were youths aged 16 to 20 years and adults aged 21 years and older.

**Main Outcomes and Measures:**

The main outcomes of interest were death, spinal cord injury, and an Injury Severity Score (ISS) greater than 25. Secondary outcomes were less severe injuries, including head injuries, crush type injuries, and fractures.

**Results:**

Among 52 745 patients with complete data, 15% were youths younger than 16 years, 13% were youths aged 16 to 20 years, 82% were male, and 47% lived in rural areas. After adjusting for covariates, the odds of dying were higher among youths aged 16 to 20 years (adjusted odds ratio [aOR], 1.64; 95% CI, 1.04-2.60) compared with those younger than 16 years. Youths aged 16 to 20 years also had higher odds of spinal cord injury (aOR, 2.72; 95% CI, 1.80-4.20) and an injury severity score greater than 25 (aOR, 1.63; 95% CI, 1.36-1.96) compared with youths 16 years of age or less. Sex-specific analyses revealed these associations were greater for male youths aged 16 to 20 years (spinal cord injury: aOR, 3.81; 95% CI, 1.38-11.10, and dying in a hospital: aOR, 4.37; 95% CI, 1.19-21.02) than female youths aged 16 to 20 years.

**Conclusions and Relevance:**

In this cross-sectional study of patients hospitalized for ATV injuries in Canada, youths aged 16 to 20 years and adults aged 21 years and older were at an increased risk of death and severe injuries compared with youths younger than 16 years. The increased risk of death and severe injuries was most evident among male patients.

## Key Points

**Question:**

Are younger patients admitted to the hospital for all-terrain vehicle (ATV)–related injuries more likely to experience worse injuries and more likely to die compared with older patients?

**Findings:**

In this cross-sectional study using a large administrative health database that included 52 745 patients hospitalized with ATV-related injuries between 2002 and 2019, older adolescents and adults, particularly male patients, were more likely to die or experience a spinal cord injury or severe trauma.

**Meaning:**

This study’s findings suggest that patients aged 16 to 20 years, particularly male individuals, admitted to the hospital for an ATV-related injury tend to present with more severe injuries and are more likely to die than younger patients.

## Teaser

This cross-sectional study investigates whether younger riders are at risk for more severe all-terrain vehicle (ATV)–related injuries compared with older riders in Canada.

## Introduction

The number of children hospitalized for all-terrain vehicle (ATV)–related injuries has increased 2- to 3-fold in Canada and the US over the past 2 decades.^1,2,3,4^ In Canada, a recent survey of 900 pediatricians revealed that a substantial percentage of youths injured while riding ATVs present to children’s hospitals with severe injuries.^5^ Studies of children and adolescents admitted to emergency departments in the US^4,6,7,8,9^ and Canada^8^ suggest that youths under 16 years of age are also more likely to experience more serious injuries and die from ATV-related injuries than older adolescents and adults. Based on this evidence, the Canadian Paediatric Society^10^ and the American Academy of Pediatrics (AAP)^11,12^ are encouraging legislation for a minimum age for ATV use for children under 16 years of age. The most recent AAP recommendations provide age-specific targets for youth and adolescents.^12^ However, the evidence for the risk of severe injury among adolescents is limited^12^ and previous work in this area suffers from common epidemiological short comings.

The limitations of previous studies of pediatric ATV-related trauma are common in pediatric injury epidemiology. Most studies rely on single-center records and are at risk of sampling bias due to parent enrollment^8^ or small regional sampling.^6,13^ Many emergency department-based studies are also limited by ascertainment bias due to missing patients with the most severe injuries who are directly admitted to intensive care.^6,8^ Many studies relied on a variety of methods and definitions to quantify injury severity,^8,13^ making it difficult to compare outcomes across studies. Additionally, very few studies have compared injury severity between male and female ATV riders, which may be different due to different riding behaviors.^14^

The current study was designed to overcome these limitations. Using administrative hospitalization data for ATV-related injuries during an 18-year period from 9 provinces in Canada, we tested the hypothesis that children and adolescents under 16 years of age would be at a greater risk of severe injuries and death following an ATV-related injury, compared with youths aged 16 years and older.

## Methods

### Data Sources and Population

To test the main hypothesis, we conducted a cross-sectional study embedded within a large national administrative registry of hospitalization records in Canada.^15^ Data were obtained for all hospitalizations for persons aged 1 to 80 years who were admitted for an ATV-related injury within the Discharge Abstract Database (DAD) housed at the Canadian Institute of Health Information.^16^ The DAD database contains diagnostic information on all admissions to acute care hospitals in Canada. Primary and secondary *International Statistical Classification of Diseases and Related Health Problems, Tenth Revision, Canada (ICD-10-CA)* revision codes and demographic information for all patients admitted to hospitals between January 1, 2002, and December 31, 2019, for an ATV-related injury in 9 provinces in Canada were included in the analysis. Hospitalization data for the province of Quebec were not available as it does not participate in the registry. Data from the 3 northern territories were not available for all years and were therefore excluded from the analyses. Patients included in this study were all patients with *ICD-10-CA* external cause of injury codes for off-road vehicle or snowmobile injuries, and they were admitted to the hospital and discharged or died following hospitalization (eTable 1 in Supplement 1). Some records had data specifying the type of ATV used during the injury; however, this was inconsistent and therefore less reliable. Patient records that did not have a primary or secondary admission diagnosis associated with an injury that would be used to calculate an injury severity score were excluded from the analyses (n = 1592). Raw data were extracted by staff at the Canadian Institute for Health Information and a password-protected database with deidentified data for age, 3-digit postal codes, sex, *ICD-10-CA*–coded injuries, outcomes, and covariates were provided to investigators for analyses. We followed the Strengthening the Reporting of Observational Studies in Epidemiology (STROBE)^17^ and Reporting of Studies Conducted Using Observational Routinely-Collected Data (RECORD)^18^ reporting guidelines to report the results of this study. This study was approved by the local ethics board at the University of Manitoba with a waiver of informed consent because deidentified data were used.

### Exposure of Interest

The primary exposure of interest was age. We categorized age as younger than 16 years at the time of the ATV injury; 16 to 20 years of age; and aged 21 years or older. An age of less than 16 years was set as the cutoff to define the age exposure for several reasons. First, 16 years of age is the minimum age to obtain a license to drive a vehicle in Canada. Second, 16 years of age is also the minimum age suggested for riding an ATV in most provinces in Canada.^19^ Lastly, data from our group^8^ and others^9^ have found differences in various injury types between adolescents stratified at 16 years of age.

### Outcomes of Interest

The main outcomes of interest were death, spinal cord injury, and an injury severity score (ISS) greater than 25.^20^ Secondary outcomes were less severe injuries, including head injuries, crush type injuries, and fractures. The cause and nature of each patient’s injury are coded according to the *ICD-10-CA*, and up to 10 injuries are coded per patient. *ICD-10-CA* codes are frequently used in administrative database studies to classify injuries and provide valid estimates of injury severity.^21^ Head injury was defined as injury to the brain, skull, scalp, or face. Spinal cord injury was defined as injury to the cervical, thoracic, or lumbar spinal cord, or cauda equina. Crush injury was defined as crush injury to the head, neck, thorax, abdomen, pelvis, upper or lower extremity, or multiple body parts. Fractures were defined as fractures to any bone of the upper or lower extremity. *ICD-10-CA* codes used to classify injury type are provided in eTable 1 in Supplement 1. Death was ascertained from discharge disposition codes. Data were not available for deaths that occurred prior to hospitalization or following hospital discharge.

ISS was derived using a crosswalk algorithm that relied on the abbreviated injury scale (AIS) to convert *ICD-10-CA* codes to an ISS score for use within Canadian registries containing *ICD-10-CA* codes.^22^ Each *ICD-10-CA* diagnosis was assigned the most conservative AIS score. In patients with *ICD-10-CA* diagnostic codes for injuries to more than 1 organ, each with a different AIS score but within the same ISS body region, the lowest AIS score was assigned. The accuracy of the algorithm was validated against abbreviated injury scores within a trauma registry, demonstrating good accuracy across various body regions and injuries (ĸ, 0.65-0.75) and performed well for youth, adolescents, and adults. The *ICD-10-CA* diagnoses that could not be assigned an accurate AIS scores were excluded from coding. ISS scores were treated as ordinal with thresholds of 15 and 25 used to classify moderate and severe injury, respectively.^23^ An ISS threshold of 25 was used to classify severe injuries because it is associated with a high probability of death.^20^

### Demographic and Covariates of Interest

The following covariates are available in the hospitalization databases that have been associated with ATV-related injuries: the province of hospital where the patient was admitted, year and month of the injury, residential postal code, and sex for the injured patient. In Canada, a postal code can be used to identify rural and urban settings. Driver (V86.5) or passenger status (V86.6) was determined from *ICD-10-CA*, which was available for a subset of patients in the registry. Data for helmet use and time spent riding an ATV prior to being hospitalized were not available within the registry.

### Statistical Analysis

Frequencies of demographic and injury characteristics were compared between age groups using χ^2^ tests. To test the study hypothesis, we performed unadjusted logistic regression analyses to determine if the odds of the outcomes of interest were higher among youths younger than age 16 years, relative to individuals aged 16 years or older. To address the risk of confounding, a logistic regression model was used that contained potential confounders (sex, province, year, urban/rural status, driving status) and adjusted odds ratios (aOR) and 95% CI were calculated to estimate statistical differences between the groups; point estimates with 95% CIs that did not overlap with 1.0 were considered statistically significant. Interaction terms were included in follow-up models to explore effect modification by sex and driving status (passenger vs driver). As less than 5% of data were missing from the available database, we did not impute missing data and no sensitivity analyses were performed. The sample was derived from eligible patients within the DAD database for the years of interest (eMethods in Supplement 1). All data were analyzed using R Studio version 1.4.1717 (R Project for Statistical Computing) while crosswalking data from an ICD-10-CA code to AIS, then to an ISS^22^ was performed using SAS version 9.4 (SAS Institute).

## Results

For these analyses, 52 745 patients had complete data; 7780 participants (15%) were under 16 years of age, 6776 (13%) were aged 16 to 20 years; 38 189 (72%) were 21 years of age or older; 43 310 (82%) were male, and 23 729 (47%) lived in a rural area (Table 1). Information on driver (n = 31 660) and passenger (n = 8799) status was available for 77% of the sample. The distribution of sex, province, and rural vs urban residence was similar across the 3 study groups.

**Table 1.** Study Participant Demographics Stratified According to Age Group

Variable	Participants, No. (%)		
	Aged <16 y	Aged 16-20 y	Aged ≥21 y
No.	7780 (15)	6776 (13)	38 189 (72)
Male	5901 (75.8)	5521 (81.5)	31 888 (83.5)
Rural	4256 (54.7)	3510 (51.8)	15 963 (41.8)
BC/AB/SK/MB	4349 (55.9)	3744 (55.2)	22 240 (58.2)
NB/NS/PEI/NF	1130 (14.5)	1049 (15.4)	5690 (14.9)
ON	2301 (29.6)	1983 (29.2)	10 259 (26.9)
Driver	4561 (58.6)	4452 (65.7)	22 647 (59.3)
Passenger	2326 (29.9)	1178 (17.4)	5295 (13.9)
Not reported	893 (11.5)	1146 (16.9)	10 247 (26.8)

Abbreviations: AB, Alberta; BC, British Columbia; MB, Manitoba; NB, New Brunswick; NF, Newfoundland & Labrador; NS, Nova Scotia; ON, Ontario; PEI, Prince Edward Island; SK, Saskatchewan.

A flowchart describing the number of patients excluded and the rationale for the final sample size used for these analyses is provided in eFigure 1 in Supplement 1. Between 2002 and 2019, 54 824 patients were admitted to the hospital for an ATV-related injury across the 9 provinces that provided data. Annual hospitalizations were relatively stable over that period ranging from a low of 748 in 2013 to a high of 1039 in 2008 (eFigure 2 in Supplement 1). We excluded data from patients with an *ICD-10-CA* code suggesting they were injured after being struck by an ATV (V86.7). An ISS score could not be calculated for 1592 patients due to missing data. There were no differences in proportions of outcomes, exposures, or covariates between those included in the analysis and the entire cohort.

The distributions of each injury type by age are provided in Table 2. The 2 most common ATV-related injuries requiring hospitalization were fractures (n = 28 581; 54.2% of hospitalizations) and head injuries (n = 5433; 10.3% of hospitalizations). There were 683 patients hospitalized with a spinal cord injury (1.3% of hospitalizations), 2226 patients had an ISS greater than 25 and 342 died in hospital (0.6% of patients).

**Table 2.** Injury Characteristics for Patients Admitted to Hospital for an All-Terrain Vehicle Injury in Canada (N = 52 745)

Variable	Patients, No. (%)					
	Aged <16 y		Aged 16-20 y		Aged ≥21 y	
No.	7780		6776		38 189	
Fractures	4711 (60.6)		3811 (56.2)		20 015 (52.4)	
Crush injuries	72 (0.9)		49 (0.7)		279 (0.7)	
Head injuries	946 (12.2)		815 (12.0)		3672 (9.6)	
Spinal cord injuries	31 (0.4)		77 (1.1)		575 (1.5)	
ISS Score (n = 51 153)						
0-15	6456 (83.0)		5367 (79.2)		28 203 (73.9)	
16-25	817 (10.5)		881 (13.0)		7163 (18.8)	
>25	214 (2.8)		304 (4.5)		1748 (4.6)	

Abbreviation: ISS, injury severity score.

In unadjusted comparisons, rates of spinal cord injuries (0.4% vs 1.1%), ISS greater than 25 (2.8% vs 4.5%), and death (0.4% vs 0.6%) were all higher among youths aged 16 to 20 years, compared with those younger than age 16 years (Table 2). Similar differences were observed when rates were compared between adults aged 21 years and older and youths aged younger than 16 years. No differences in the rates of severe injuries were observed between youths aged 16 to 20 years and adults aged 21 years and older. The unadjusted odds of different injury types and outcomes are presented in eFigure 3 and eTable 2 in Supplement 1.

**Figure 1.**
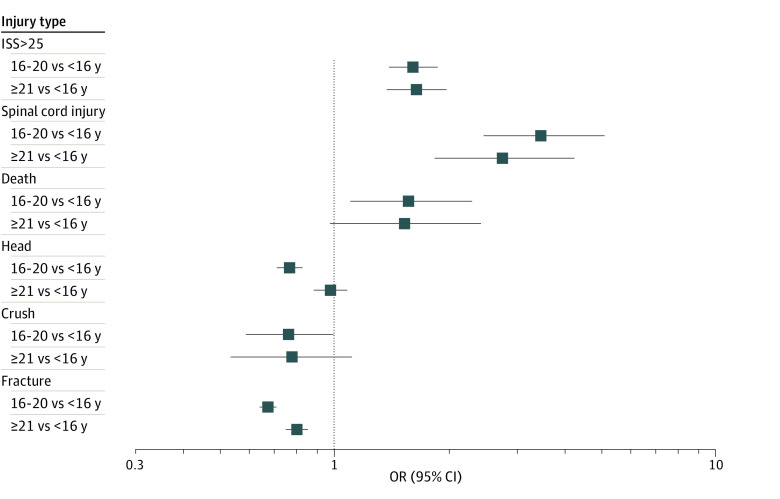
Adjusted Odds of ATV-Related Injuries for Groups of Individuals Aged 16 to 20 Years and 21 Years and Older Compared With Those Younger Than 16 Years See eTable 2 in Supplement 1 for corresponding numerical data.

The odds of different injury outcomes, after adjusting for covariates, are presented in Figure 1 and eTable 2 in Supplement 1. Compared with youths younger than 16 years of age, youths aged 16 to 20 years and adults aged 21 years or older were more likely to be hospitalized for a spinal cord injury (aged 16 to 20 years: aOR, 2.72 [95% CI, 1.80-4.20]; 21 years or older: aOR, 3.38 [95% CI, 2.71-5.60]), have an ISS score greater than 25 (aged 16 to 20 years: aOR, 1.63 [95% CI, 1.36-1.96]; 21 years or older: aOR, 1.63 [95% CI, 1.41-1.89]), and were more likely to die from an ATV-related injury (aged 16 to 20 years: aOR, 1.64 [95% CI, 1.04-2.60]; 21 years or older: aOR, 1.75 [95% CI, 1.23-2.57]). These point estimates and 95% CIs for age-specific differences in mortality and spinal cord injury remained similar when analyses were repeated when the 1592 patients for whom we could not calculate an ISS score were added to the cohort (n = 52 745).

**Figure 2.**
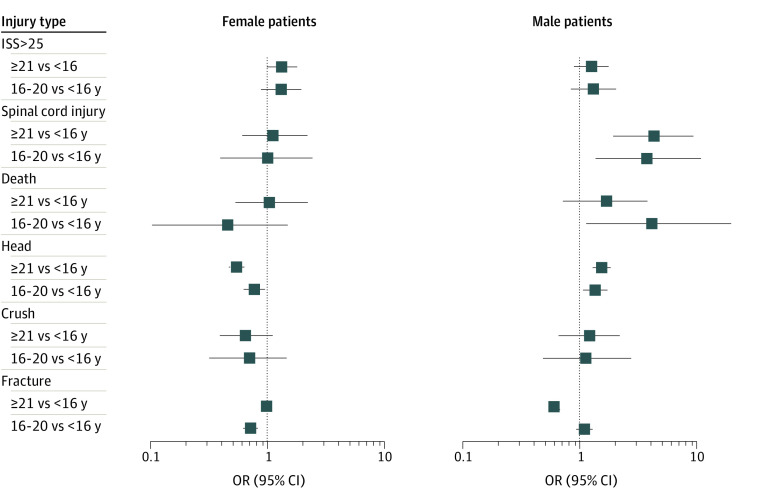
Sex-Specific Odds of Various Injuries for Groups of Individuals Aged 16 to 20 Years and 21 Years and Older Admitted to Hospital for an ATV-Related Injury Compared With Those Younger Than 16 Years See eTable 2 in Supplement 1 for corresponding numerical data.

Results of sensitivity analyses are presented in eFigures 2 and 4 and eTables 3 and 4 in Supplement 1. Interaction terms suggested sex differences in the associations between age and the odds of a head, spinal cord, and fracture injury as well as death (eTable 3 in Supplement 1; *P* < .1 for all interactions). We therefore repeated the analyses for male and female patients separately for each study outcome (Figure 2). Among female patients, fully adjusted models revealed no statistically significant difference in the odds of a spinal cord injury or death based on age. However, adults aged 21 years or older experienced significantly higher odds of an ISS greater than 25 (aOR, 1.47 [95% CI, 1.10-2.01]) compared with youths aged 16 years or younger. Among male patients compared with youths aged younger than 16 years, youths aged 16 to 20 years and adults aged 21 years or older had higher odds of a spinal cord injury (youths aged 16 to 20 years: aOR, 3.81 [95% CI, 1.38-11.10]; and adults aged 21 year or older: aOR, 4.40 [95% CI, 1.96-9.59]), and dying in hospital (youths aged 16 to 20 years: aOR, 4.37 [95% CI, 1.19-21.02]; adults aged 21 years or older: aOR, 1.75 [95% CI, 0.74-3.93]). After adjusting for region, sex, province, and year of the injury, youths aged 16 to 20 years (aOR, 0.80 [95% CI, 0.74-0.85]) and adults (aOR, 0.66 [95% CI, 0.63-0.70]) were less likely to be hospitalized for fractures compared with youths younger than 16 years of age. Relative to youths younger than 16 years of age, the odds of sustaining a head injury were lower among female individuals aged 16 to 20 years and 21 years or older, but higher among male individuals aged 16 to 20 years and 21 years or older. Among individuals aged 16 to 20 years and 21 years or older, passengers were significantly more likely than drivers to experience spinal cord injuries or sustain an injury with an ISS greater than 25 (eFigure 4 in Supplement 1).

## Discussion

Among patients hospitalized for ATV injuries in Canada, the odds of severe injuries and death were higher among youths aged 16 to 20 years and adults aged 21 years or older compared with youths younger than 16 years of age. The increased odds of severe injury and mortality among those aged 16 to 20 years was particularly evident among male individuals.

Calls for age restrictions for ATV frequently argue that younger children are at a substantial risk for injuries and death when operating these vehicles.^10,19^ We found that in Canada, between 2002 and 2019, approximately 8000 youths under 16 years of age and approximately 7000 youths aged 16 to 20 years of age were admitted to a hospital for an ATV-related injury. These data are higher than the rates of hospitalizations for ATV injuries in Canada between 1996 and 2004. The current data support previous work that young adults and adolescents were the most frequently hospitalized.^24^ Age-specific rates of hospitalizations for ATV injuries in Canada have only been reported for specific provinces^25,26^; and none examined injury rates compared with adults. A previous cross-sectional study by our group, using a convenience sample of pediatric patients seen in emergency departments in Canada, found that younger children are more likely to experience a head injury and fractures, relative to older adolescents.^8^ Studies in the US suggest that children admitted to the hospital following an ATV injury were at higher risk of severe trauma relative to adults, however injury severity itself was not examined.^27^ A small case-control study in the US (n = 133 hospitalized patients), found that youths under 15 years of age displayed a 12-fold higher odds of being hospitalized, compared with adults over 45 years of age. Other studies suggest the opposite,^26^ with the risk of an ATV-related fatality highest among youths aged 16 to 19 years.^9^ The data presented here support the latter, demonstrating that, on a population-level, youths aged 16 to 20 years admitted to a hospital in Canada were more likely to sustain spinal cord injuries, severe trauma, or die following a hospitalization for an ATV injury, compared with youths younger than 16 years of age. These data highlight the need to include older adolescents and young adults in public health messaging aimed at reducing ATV-related injuries.

Boys and men are 2 to 3 times more likely to be admitted to a hospital for an ATV-related injury compared with girls and women.^4,8,28^ The recent AAP guidelines recognize that boys are at a greater risk of crashes and injuries,^12^ likely due to increased risk-taking behaviors.^29^ In the US between 2000 and 2005, boys and men were also more likely to die from an ATV-related injury, compared with girls and women.^30^ In Canada, rates of admissions to emergency departments for ATV-related injuries are 2- to 4-fold higher in boys than girls.^8,26^ We observed that 75% to 84% of patients under 20 years of age admitted to hospitals in Canada for ATV-related injuries are boys. Among male youths, but not female youths, the risks of death and spinal cord injury following an ATV-related injury were 3- to 4-fold higher in those aged 16 to 20 years compared with youths under 16 years of age. These trends could be explained by more frequent ATV use and riskier riding behaviors among male youths.^14^ Overall, these data suggest that the burden of ATV-related hospitalizations and severe injuries is higher among male youths aged 16 to 20 years, relative to female youths in the same age group, and this information could inform targeted public health prevention strategies.

### Limitations

This study has limitations. First, as with many large administrative databases, several variables that contribute to the risk of injury severity were not available, particularly, lifestyle-related factors including ATV exposure time (ie, hours of riding), use drugs or alcohol use at the time of injury, parental supervision, risky riding behaviors and purpose of ATV use (transportation vs recreational use). A better understanding of lifestyle factors and risk-seeking behaviors could help explain the sex-specific differences we observed in this descriptive study. Second, a notable proportion of data were missing for individuals living in the Northern territories where ATV use is very high and more important covariates including driver status,^8^ helmet use,^8,31^ and/or effect modifiers, such as type of ATV.^32^ It is unclear if differences in these covariates contributed to the differences in injury severity observed here. Additionally, data from patients who were not seen in the hospital, were only seen in an emergency department or either died in the emergency department or outside the hospital were not included in the analysis and could potentially influence the injury rates and trends reported here.

## Conclusions

Individuals aged 16 years or older are at a greater risk of death and severe ATV-related injuries compared with those younger than 16 years of age. The increased risk of death and severe injuries among youths aged 16 to 20 years is particularly evident among male youths compared with female youths. Children and adolescents younger than 16 years of age are more likely to experience less severe injuries, including head injuries, fractures, and crush injuries. These results suggest that in addition to children and young adolescents, policies and public health messaging to reduce injuries and hospitalizations should target ATV riders within the older adolescent and young adult age range, and potentially targeted toward male individuals.

## Notes

Supplement 1. **eFigure 1.** A Flowchart Containing the Steps of Cohort Development **eFigure 2.** Injury Rates Over Time Stratified by (A) Mild Injuries and (B) Severe Injuries and Death **eFigure 3.** Unadjusted Odds Ratios of Main Injury Outcomes for Adolescents and Adults, Compared to Youth Admitted to Hospital for an ATV-Related Injury **eFigure 4.** Adjusted Odds Ratios of Main Injury Outcomes for Adolescents and Adults Admitted to Hospital for an ATV-Related Injury, Stratified by Driving Status (Models Included Year, Province, Urban/Rural Status, Sex, Driving Status and the Age by Driving Status Interaction) **eTable 1.** ICD Codes Used to Determine Injury Type and Calculate Injury Severity Scores **eTable 2.** Odds of Main Injury Outcomes for Older and Younger Patients (Reference Group: Age <16) Admitted to Hospital for an ATV-Related Injury From Five Models **eTable 3.** Results of Age and Sex Interaction Terms from Models Adjusted for Covariates, Driving Status, and Age-Sex Interaction **eTable 4.** Results of Age and Driving Status Interaction Term from the Model Adjusted for Covariates, Driving Status, and Age-Driving Status Interaction **eFigure 5.** Unadjusted Odds Ratios of Main Injury Outcomes for Adolescents and Adults, Compared to Youth Admitted to Hospital for an ATV-Related Injury (n=52,745; ISS Data n= 51,153) **eFigure 6.** Adjusted Odds Ratios of Main Injury Outcomes for Adolescents and Adults Admitted to Hospital for an ATV-Related Injury, Stratified by Driving Status (Models Included Year, Province, Urban/Rural Status, Sex, Driving Status and the Age by Driving Status Interaction) **eTable 5.** Odds of Main Injury Outcomes for Older and Younger Patients (Reference Group: Age <16) Admitted to Hospital for an ATV-Related Injury from Five Models **eTable 6.** Results of Age and Sex Interaction Terms from Models Adjusted for Covariates, Driving Status, and Age-Sex Interaction **eTable 7.** Results of Age and Driving Status Interaction Term from the Model Adjusted for Covariates, Driving Status, and Age-Driving Status Interaction **eMethods.** Codes Used for All Analyses Described in the Analytical Section

Supplement 2.
Data Sharing Statement
