# Supplementary material for: Severity of All-Terrain Vehicle–Related Injuries by Age in Canada, 2002-2019
Source: JAMA Netw Open. 2023 May 31;6(5):e2316060. doi: 10.1001/jamanetworkopen.2023.16060 (PMC10233422; doi:10.1001/jamanetworkopen.2023.16060)
Supplement: Supplement 1. — eFigure 1. A Flowchart Containing the Steps of Cohort Development eFigure 2. Injury Rates Over Time Stratified by (A) Mild Injuries and (B) Severe Injuries and Death eFigure 3. Unadjusted Odds Ratios of Main Injury Outcomes for Adolescents and Adults, Compared to Youth Admitted to Hospital for an ATV-Related Injury eFigure 4. Adjusted Odds Ratios of Main Injury Outcomes for Adolescents and Adults Admitted to Hospital for an ATV-Related Injury, Stratified by Driving Status (Models Included Year, Province, Urban/Rural Status, Sex, Driving Status and the Age by Driving Status Interaction) eTable 1. ICD Codes Used to Determine Injury Type and Calculate Injury Severity Scores eTable 2. Odds of Main Injury Outcomes for Older and Younger Patients (Reference Group: Age <16) Admitted to Hospital for an ATV-Related Injury From Five Models eTable 3. Results of Age and Sex Interaction Terms from Models Adjusted for Covariates, Driving Status, and Age-Sex Interaction eTable 4. Results of Age and Driving Status Interaction Term from the Model Adjusted for Covariates, Driving Status, and Age-Driving Status Interaction eFigure 5. Unadjusted Odds Ratios of Main Injury Outcomes for Adolescents and Adults, Compared to Youth Admitted to Hospital for an ATV-Related Injury (n=52,745; ISS Data n= 51,153) eFigure 6. Adjusted Odds Ratios of Main Injury Outcomes for Adolescents and Adults Admitted to Hospital for an ATV-Related Injury, Stratified by Driving Status (Models Included Year, Province, Urban/Rural Status, Sex, Driving Status and the Age by Driving Status Interaction) eTable 5. Odds of Main Injury Outcomes for Older and Younger Patients (Reference Group: Age <16) Admitted to Hospital for an ATV-Related Injury from Five Models eTable 6. Results of Age and Sex Interaction Terms from Models Adjusted for Covariates, Driving Status, and Age-Sex Interaction eTable 7. Results of Age and Driving Status Interaction Term from the Model Adjusted for Covariates, Driving Status, and Age-Driving [file jamanetwopen-e2316060-s001.pdf]

Supplemental Online Content

MacDougall W, Jiang X, Sobhan S, et al. Severity of all-terrain vehicle–related injuries by age in Canada, 2002-2019. *JAMA Netw Open.* 2023;6(5):e2316060. doi:10.1001/jamanetworkopen.2023.16060

eFigure 1. A Flowchart Containing the Steps of Cohort Development

eFigure 2. Injury Rates Over Time Stratified by (A) Mild Injuries and (B) Severe Injuries and Death

eFigure 3. Unadjusted Odds Ratios of Main Injury Outcomes for Adolescents and Adults, Compared to Youth Admitted to Hospital for an ATV-Related Injury

eFigure 4. Adjusted Odds Ratios of Main Injury Outcomes for Adolescents and Adults Admitted to Hospital for an ATV-Related Injury, Stratified by Driving Status (Models Included Year, Province, Urban/Rural Status, Sex, Driving Status and the Age by Driving Status Interaction)

eTable 1. ICD Codes Used to Determine Injury Type and Calculate Injury Severity Scores

eTable 2. Odds of Main Injury Outcomes for Older and Younger Patients (Reference Group: Age <16) Admitted to Hospital for an ATV-Related Injury From Five Models

eTable 3. Results of Age and Sex Interaction Terms from Models Adjusted for Covariates, Driving Status, and Age-Sex Interaction

eTable 4. Results of Age and Driving Status Interaction Term from the Model Adjusted for Covariates, Driving Status, and Age-Driving Status Interaction

eFigure 5. Unadjusted Odds Ratios of Main Injury Outcomes for Adolescents and Adults, Compared to Youth Admitted to Hospital for an ATV-Related Injury (n=52,745; ISS Data n= 51,153)

eFigure 6. Adjusted Odds Ratios of Main Injury Outcomes for Adolescents and Adults Admitted to Hospital for an ATV-Related Injury, Stratified by Driving Status (Models Included Year, Province, Urban/Rural Status, Sex, Driving Status and the Age by Driving Status Interaction)

eTable 5. Odds of Main Injury Outcomes for Older and Younger Patients (Reference Group: Age <16) Admitted to Hospital for an ATV-Related Injury from Five Models

eTable 6. Results of Age and Sex Interaction Terms from Models Adjusted for Covariates, Driving Status, and Age-Sex Interaction

© 2023 MacDougall W et al. *JAMA Network Open.*

eTable 7. Results of Age and Driving Status Interaction Term from the Model Adjusted for Covariates, Driving Status, and Age-Driving Status Interaction

eMethods. Codes Used for All Analyses Described in the Analytical Section

This supplemental material has been provided by the authors to give readers additional information about their work.

© 2023 MacDougall W et al. *JAMA Network Open.*

eFigure 1: A Flowchart containing the steps of cohort development

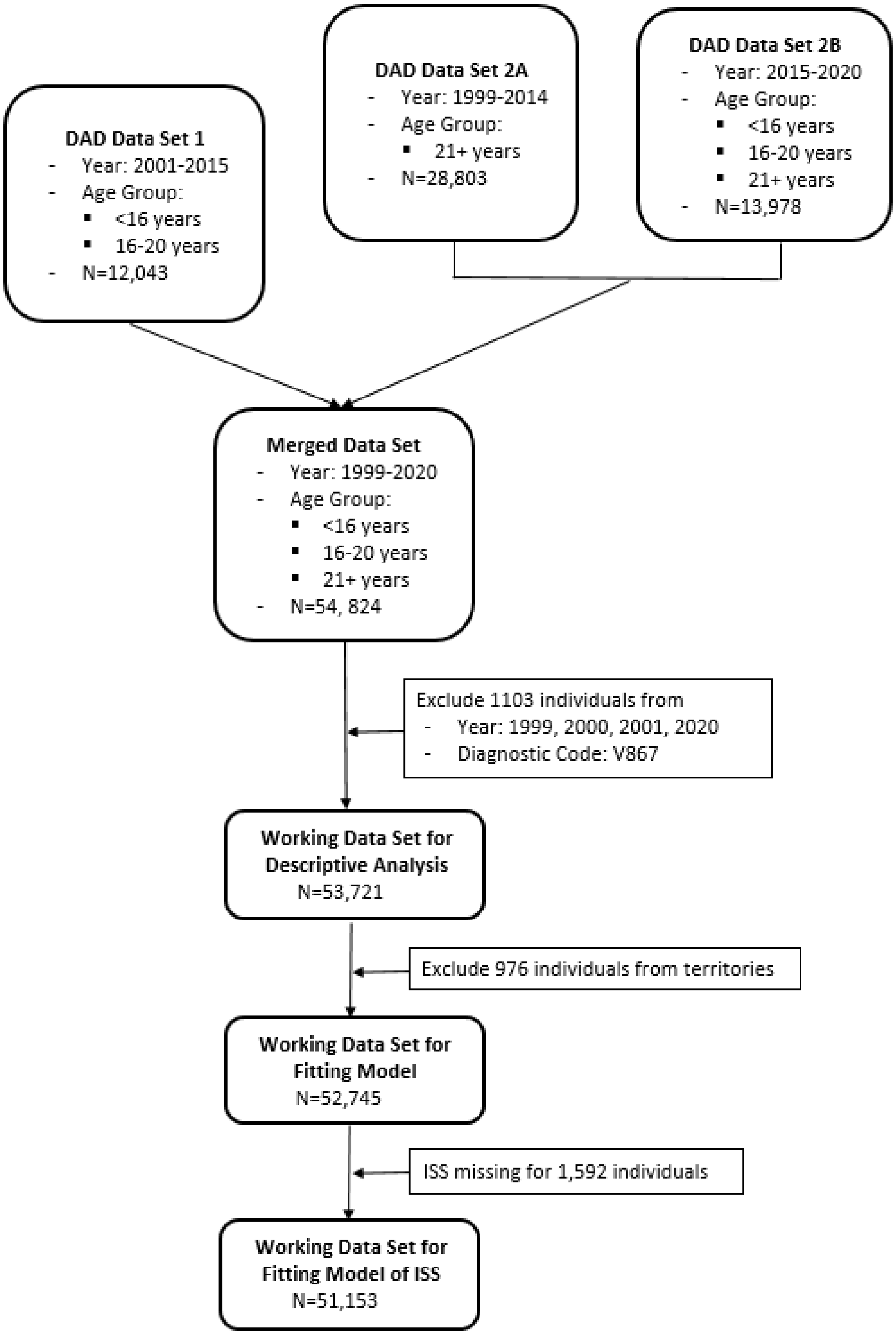

ISS = injury severity score, ICD code V867 refers to ICD-10 code V86.7 – person struck by an ATV

© 2023 MacDougall W et al. *JAMA Network Open.*

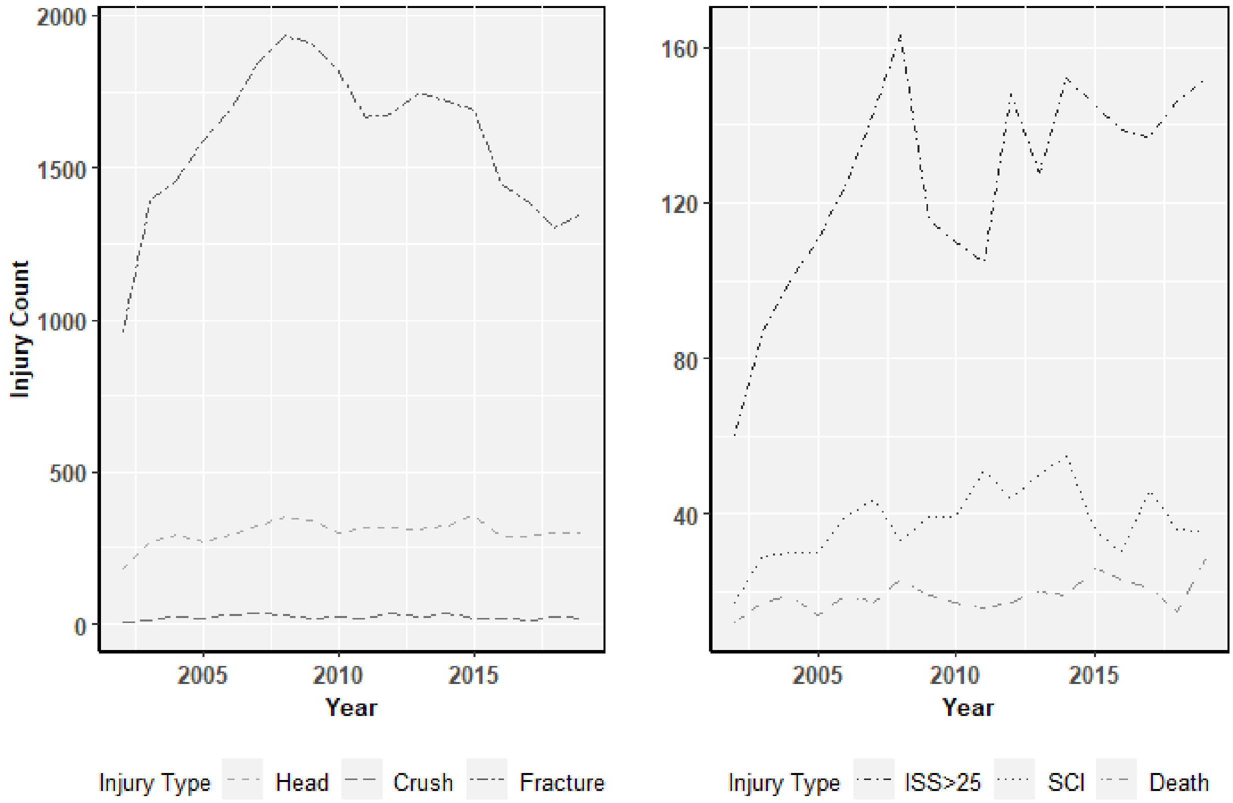

eFigure 2. Injury rates over time stratified by (A) mild injuries and (B) severe injuries and death.

ISS = Injury severity score; SCI = Spinal Cord Injury

© 2023 MacDougall W et al. *JAMA Network Open.*

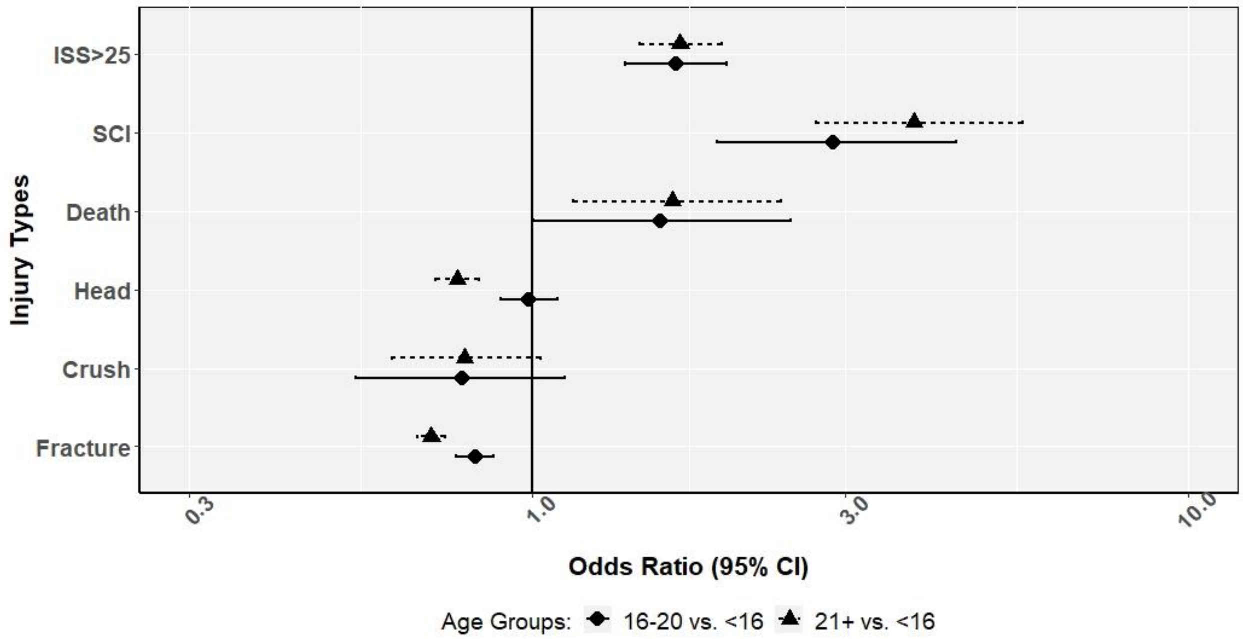

eFigure 3: Unadjusted odds ratios of main injury outcomes for adolescents and adults, compared to youth admitted to hospital for an ATV-related injury. ISS = injury severity score; SCI = spinal cord injury

© 2023 MacDougall W et al. *JAMA Network Open.*

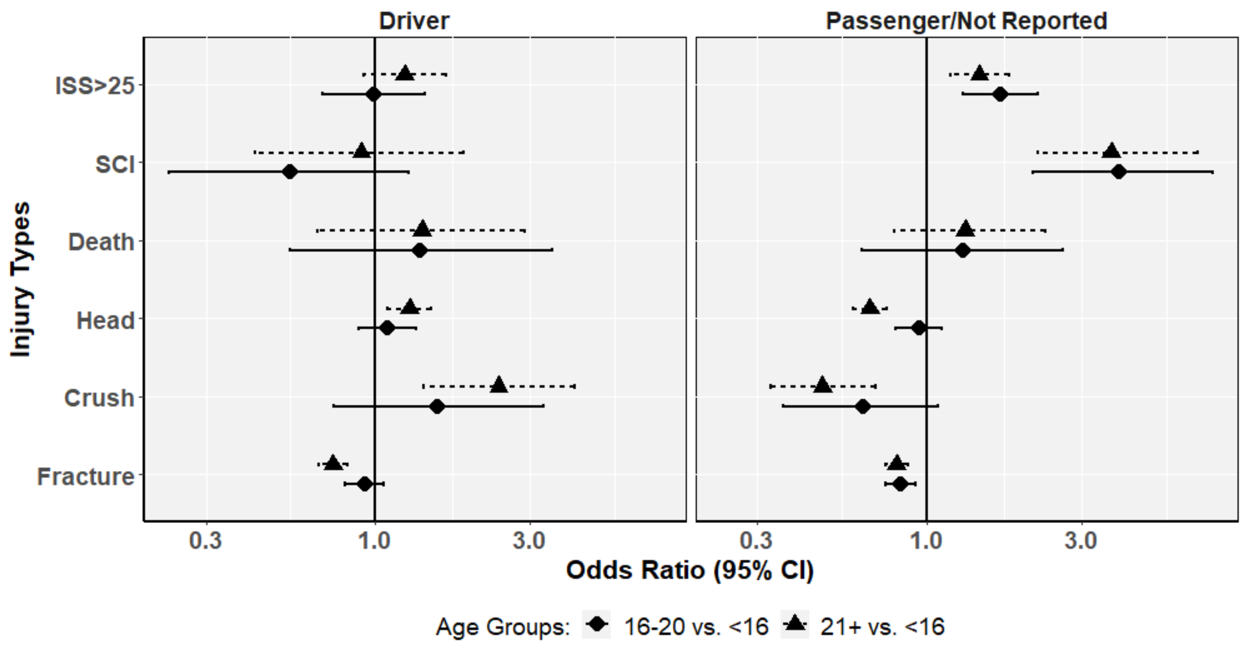

Figure S4. Adjusted odds ratios of main injury outcomes for adolescents and adults admitted to hospital for an ATV-related injury, stratified by driving status (models included year, province, urban/rural status, sex, driving status and the age by driving status interaction).

© 2023 MacDougall W et al. *JAMA Network Open.*

eTable 1. ICD codes used to determine injury type and calculate injury severity scores

Injury Type	ICD-10-CA
ATV Injury	V86.0, V86.08-V86.98
Head injury (intracranial)	S060-S069
Extremity fractures	S420-S429, S520-S529, S620-S629<br>S720-S729, S820-S829, S920-S929<br>T022-T027, T10, T12
Spinal cord injury (SCI with or without spinal fracture, cauda equina syndrome)	S140-S14.19, S240-S24.19, S340-S34.19, S343-S34.38
Crush-type injury	S070-S079, S170-S179, S280, S380-S381, S470-S478, S570-S579, S670-S678, S770-S772, S870-S878, S970-S978, T040-T049

© 2023 MacDougall W et al. *JAMA Network Open.*

eTable 2. Odds of main injury outcomes for older and younger patients (reference Group: age <16) admitted to hospital for an ATV-related injury from five models. ISS = injury severity score; SCI = spinal cord injury (n=51, 153).

Injury	Model	Category	Age Group	Odds Ratio (CI)	P-value
Head	M1	Unadjusted	16-20	0.99 (0.89, 1.09)	0.808
			21+	0.77 (0.71, 0.83)	<0.001
	M2	Adjusted	16-20	0.99 (0.89, 1.09)	0.71
			21+	0.77(0.71, 0.83)	<0.001
	M3	Female	16-20	0.78 (0.63, 0.96)	0.020
			21+	0.55 (0.47, 0.64)	<0.001
		Male	16-20	1.38 (1.09, 1.76)	0.009
			21+	1.57 (1.31, 1.88)	<0.001
	M4	Driver	16-20	0.94 (0.80, 1.11)	0.487
			21+	0.67 (0.59, 0.75)	<0.001
		Passenger/Not Reported	16-20	1.09 (0.89, 1.34)	0.419
			21+	1.27 (1.09, 1.49)	0.002
Fracture	M1	Unadjusted	16-20	0.82 (0.77, 0.87)	<0.001
			21+	0.70 (0.67, 0.74)	<0.001
	M2	Adjusted	16-20	0.80 (0.75, 0.86)	<0.001
			21+	0.68 (0.64, 0.71)	<0.001
	M3	Female	16-20	0.72 (0.62, 0.83)	<0.001
			21+	0.99 (0.89, 1.10)	0.850
		Male	16-20	1.12 (0.95, 1.31)	0.186
			21+	0.61 (0.54, 0.69)	<0.001
	M4	Driver	16-20	0.83 (0.75, 0.93)	<0.001
			21+	0.81 (0.75, 0.87)	<0.001
			16-20	0.93 (0.81, 1.06)	0.277

© 2023 MacDougall W et al. *JAMA Network Open.*

		Passenger/Not Reported	21+	0.74 (0.67, 0.82)	<0.001
Crush	M1	Unadjusted	16-20	0.78 (0.54, 1.12)	0.181
			21+	0.79 (0.61, 1.03)	0.073
	M2	Adjusted	16-20	0.78 (0.54, 1.12)	0.185
			21+	0.77 (0.59, 1.00)	0.047
	M3	Female	16-20	0.71 (0.32, 1.47)	0.371
			21+	0.65 (0.39, 1.12)	0.106
		Male	16-20	1.15 (0.49, 2.81)	0.755
			21+	1.24 (0.67, 2.25)	0.486
	M4	Driver	16-20	0.64 (0.36, 1.09)	0.108
			21+	0.48 (0.33, 0.70)	<0.001
		Passenger/Not Reported	16-20	1.56 (0.75, 3.32)	0.242
			21+	2.40 (1.41, 4.11)	0.001
Death	M1	Unadjusted	16-20	1.57 (1.00, 2.48)	0.050
			21+	1.63 (1.16, 2.39)	0.008
	M2	Adjusted	16-20	1.54 (0.98, 2.44)	0.062
			21+	1.58 (1.11, 2.31)	0.015
	M3	Female	16-20	0.46 (0.10, 1.51)	0.238
			21+	1.05 (0.53, 2.24)	0.902
		Male	16-20	4.23 (1.16, 20.35)	0.042
			21+	1.73 (0.73, 3.89)	0.195
	M4	Driver	16-20	1.29 (0.63, 2.63)	0.477
			21+	1.31 (0.79, 2.32)	0.316
		Passenger/Not Reported	16-20	1.37 (0.54, 3.50)	0.507
			21+	1.39 (0.67, 2.90)	0.376
ISS>25	M1	Unadjusted	16-20	1.65 (1.38, 1.98)	<0.001
			21+	1.68 (1.46, 1.95)	<0.001

© 2023 MacDougall W et al. *JAMA Network Open.*

	M2	Adjusted	16-20	1.65 (1.38, 1.98)	<0.001
			21+	1.62 (1.40, 1.88)	<0.001
	M3	Female	16-20	1.32 (0.89, 1.97)	0.168
			21+	1.33 (1.00, 1.81)	0.058
		Male	16-20	1.33 (0.85, 2.09)	0.206
			21+	1.29 (0.91, 1.80)	0.148
	M4	Driver	16-20	1.68 (1.29, 2.20)	<0.001
			21+	1.45 (1.18, 1.80)	<0.001
		Passenger/Not Reported	16-20	0.99 (0.69, 1.42)	0.959
			21+	1.23 (0.92, 1.65)	0.163
SCI	M1	Unadjusted	16-20	2.87 (1.91, 4.43)	<0.001
			21+	3.81 (2.71, 5.60)	<0.001
	M2	Adjusted	16-20	2.78 (1.85, 4.29)	<0.001
			21+	3.50 (2.48, 5.14)	<0.001
	M3	Female	16-20	1.01 (0.40, 2.45)	0.979
			21+	1.12 (0.61, 2.22)	0.725
		Male	16-20	3.85 (1.39, 11.21)	0.011
			21+	4.42 (1.97, 9.64)	<0.001
	M4	Driver	16-20	3.91 (2.13, 7.66)	<0.001
			21+	3.71 (2.21, 6.83)	<0.001
		Passenger/Not Reported	16-20	0.55 (0.23, 1.27)	0.161
			21+	0.90 (0.43, 1.87)	0.789

Model 1: Unadjusted model;
Model 2: Adjusted for Covariates and Driving Status;
Model 3: Adjusted for Covariates, Driving Status and Age-Sex Interaction;
Model 4: Adjusted for Covariates, Driving Status and Age-Driving Status Interaction.
Comparison group= Youths under 16 years of age for all models.

© 2023 MacDougall W et al. *JAMA Network Open.*

eTable 3: Results of Age and Sex Interaction Terms from Models Adjusted for Covariates, Driving Status, and Age-Sex Interaction.

Injury	Deviance	P-Value
Head	24.35	<0.001
SCI	12.15	0.002
Fracture	118.22	<0.001
Crush	0.48	0.786
Mortality	4.81	0.090
ISS	2.22	0.329

SCI = Spinal Cord Injury; ISS = Injury severity score

eTable 4: Results of Age and Driving Status Interaction Term from the Model Adjusted for Covariates, Driving Status, and Age-Driving Status Interaction.

Injury	Deviance	P-Value
Head	10.76	0.005
SCI	4.31	0.116
Fracture	43.55	<0.001
Crush	11.06	0.004
Mortality	0.79	0.675
ISS	4.13	0.127

SCI = Spinal Cord Injury; ISS = Injury severity score

© 2023 MacDougall W et al. *JAMA Network Open.*

eFigure 5: Unadjusted odds ratios of main injury outcomes for adolescents and adults, compared to youth admitted to hospital for an ATV-related injury (n=52,745; ISS data n= 51,153). ISS = injury severity score; SCI = spinal cord injury

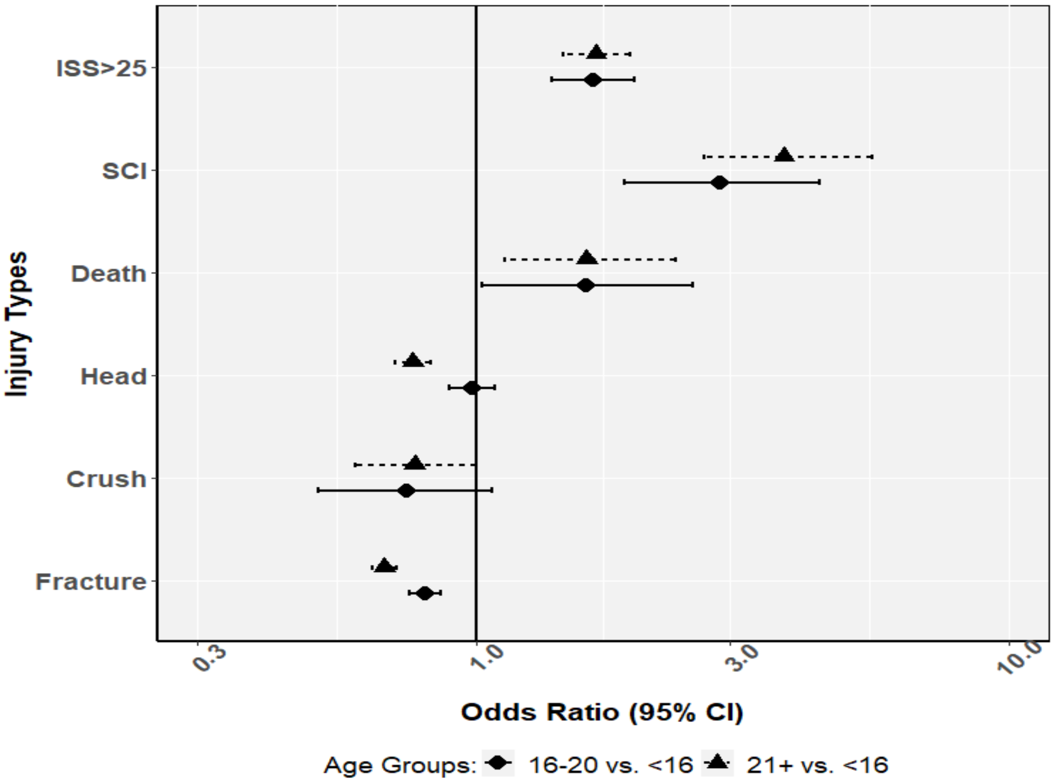

© 2023 MacDougall W et al. *JAMA Network Open.*

eFigure 6. Adjusted odds ratios of main injury outcomes for adolescents and adults admitted to hospital for an ATV-related injury, stratified by driving status (models included year, province, urban/rural status, sex, driving status and the age by driving status interaction). (n=52,745; ISS data n= 51,153)

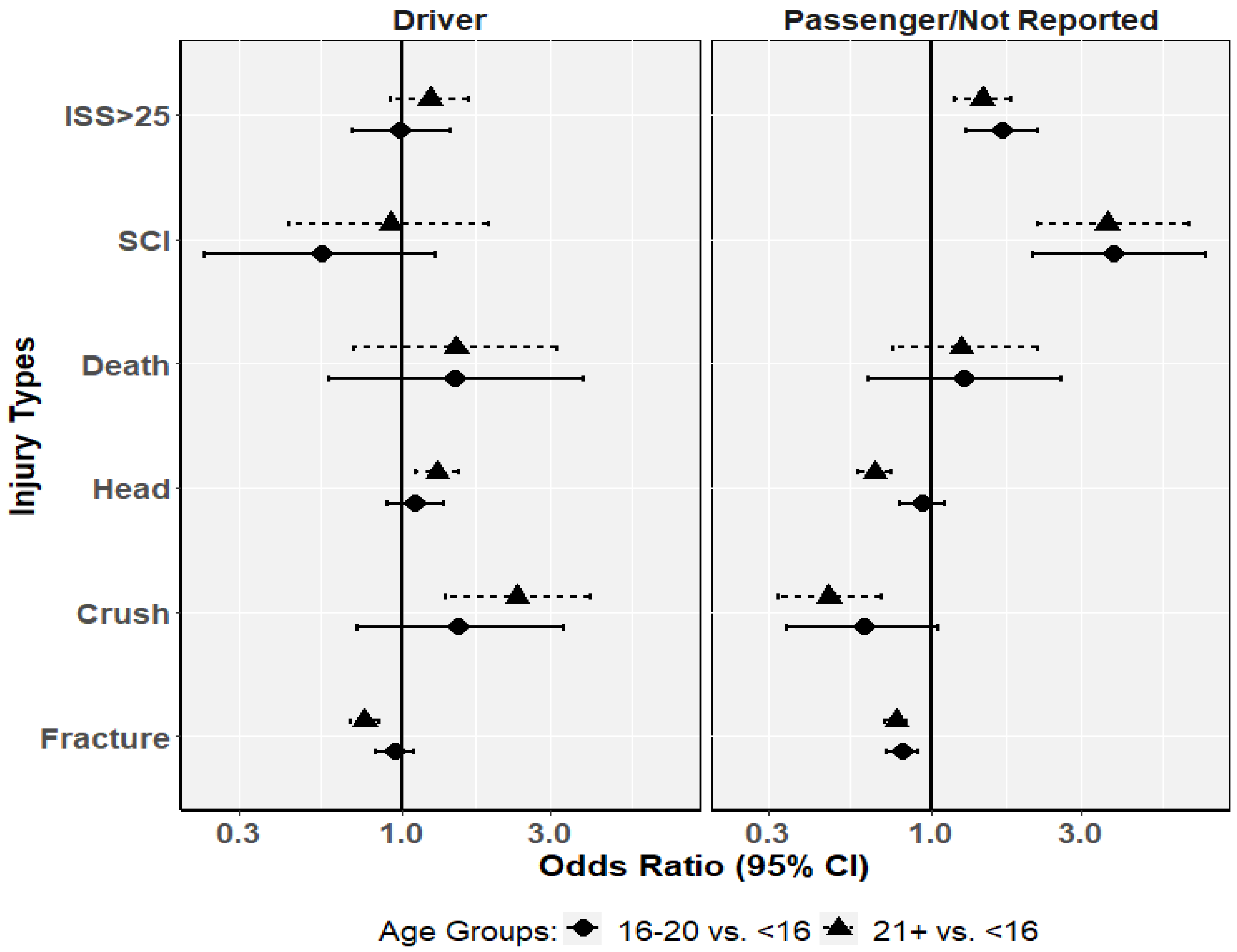

SCI = Spinal Cord Injury; ISS = Injury severity score

© 2023 MacDougall W et al. *JAMA Network Open.*

eTable 5: Odds of main injury outcomes for older and younger patients (reference Group: age <16) admitted to hospital for an ATV-related injury from five models. ISS = injury severity score; SCI = spinal cord injury. (n=52,745)

Injury	Model	Category	Age Group	Odds Ratio (CI)	P-value
Head	M1	Unadjusted	16-20	0.98 (0.89, 1.09)	0.726
			21+	0.76 (0.70, 0.82)	<0.001
	M2	Adjusted	16-20	0.98 (0.89, 1.09)	0.717
			21+	0.76 (0.71, 0.83)	<0.001
	M3	Female	16-20	0.78 (0.63, 0.96)	0.021
			21+	0.54 (0.46, 0.63)	<0.001
		Male	16-20	1.38 (1.08, 1.75)	0.010
			21+	1.59 (1.33, 1.90)	<0.001
	M4	Driver	16-20	0.93 (0.79, 1.10)	0.400
			21+	0.66 (0.58, 0.74)	<0.001
		Passenger/Not Reported	16-20	1.10 (0.90, 1.36)	0.348
			21+	1.30 (1.11, 1.52)	0.001

© 2023 MacDougall W et al. *JAMA Network Open.*

Fracture	M1	Unadjusted	16-20	0.80 (0.75, 0.86)	<0.001
			21+	0.67 (0.64, 0.71)	<0.001
	M2	Adjusted	16-20	0.79 (0.74, 0.84)	<0.001
			21+	0.65 (0.62, 0.69)	<0.001
	M3	Female	16-20	0.71 (0.61, 0.83)	<0.001
			21+	0.96 (0.86, 1.07)	0.473
		Male	16-20	1.10 (0.93, 1.30)	0.255
			21+	0.61 (0.54, 0.69)	<0.001
	M4	Driver	16-20	0.80 (0.72, 0.90)	<0.001
			21+	0.77 (0.71, 0.83)	<0.001
		Passenger/Not Reported	16-20	0.95 (0.83, 1.10)	0.484
			21+	0.76 (0.68, 0.84)	<0.001
Crush	M1	Unadjusted	16-20	0.74 (0.51, 1.07)	0.111
			21+	0.77 (0.59, 1.00)	0.046

© 2023 MacDougall W et al. *JAMA Network Open.*

	M2	Adjusted	16-20	0.74 (0.51, 1.07)	0.113
			21+	0.74 (0.57, 0.98)	0.029
	M3	Female	16-20	0.64 (0.28, 1.36)	0.264
			21+	0.59 (0.35, 1.01)	0.048
		Male	16-20	1.22 (0.51, 3.11)	0.657
			21+	1.36 (0.73, 2.50)	0.319
	M4	Driver	16-20	0.61 (0.34, 1.05)	0.084
			21+	0.47 (0.32, 0.69)	<0.001
		Passenger/Not Reported	16-20	1.52 (0.72, 3.29)	0.279
			21+	2.35 (1.38, 4.04)	0.002
Death	M1	Unadjusted	16-20	1.61 (1.03, 2.56)	0.040
			21+	1.61 (1.13. 2.37)	0.011
	M2	Adjusted	16-20	1.58 (1.00, 2.51)	0.050
			21+	1.55 (1.08, 2.29)	0.021

© 2023 MacDougall W et al. *JAMA Network Open.*

	M3	Female	16-20	0.46 (0.10, 1.51)	0.239
			21+	0.94 (0.48, 2.02)	0.865
		Male	16-20	4.39 (1.20, 21.20)	0.037
			21+	1.94 (0.81, 4.42)	0.122
	M4	Driver	16-20	1.27 (0.62, 2.59)	0.506
			21+	1.24 (0.75, 2.20)	0.423
		Passenger/Not Reported	16-20	1.48 (0.58, 3.82)	0.412
			21+	1.49 (0.71, 3.15)	0.291
SCI	M1	Unadjusted	16-20	2.86 (1.90, 4.41)	<0.001
			21+	3.78 (2.68, 5.55)	<0.001
	M2	Adjusted	16-20	2.77 (1.84, 4.27)	<0.001
			21+	3.84 (2.46, 5.11)	<0.001
	M3	Female	16-20	1.02 (0.40, 2.47)	0.969
			21+	1.11 (0.61, 2.20)	0.748

© 2023 MacDougall W et al. *JAMA Network Open.*

		Male	16-20	3.80 (1.38, 11.10)	0.011
			21+	4.44 (1.98. 9.67)	<0.001
	M4	Driver	16-20	3.86 (2.10, 7.57)	<0.001
			21+	3.65 (2.17, 6.72)	<0.001
		Passenger/Not Reported	16-20	0.55 (0.23, 1.29)	0.171
			21+	0.92 (0.43, 1.91)	0.824

Model 1: Unadjusted model;

Model 2: Adjusted for Covariates and Driving Status;

Model 3: Adjusted for Covariates, Driving Status and Age-Sex Interaction;

Model 4: Adjusted for Covariates, Driving Status and Age-Driving Status Interaction.

SCI = Spinal Cord Injury; ISS = Injury severity score

Comparison group = youths under 16 years of age.

© 2023 MacDougall W et al. *JAMA Network Open.*

eTable 6. Results of Age and Sex Interaction Terms from Models Adjusted for Covariates, Driving Status, and Age-Sex Interaction.

Injury	Deviance	P-Value
Head	25.48	<0.001
SCI	12.20	0.002
Fracture	113.54	<0.001
Crush	0.98	0.613
Mortality	0.08	0.079

eTable 7. Results of Age and Driving Status Interaction Term from the Model Adjusted for Covariates, Driving Status, and Age-Driving Status Interaction.

Injury	Deviance	P-Value
Head	12.15	0.002
SCI	4.32	0.115
Fracture	36.92	<0.001
Crush	10.38	0.006
Mortality	1.12	0.571

SCI = spinal Cord Injury; ISS = Injury severity score

© 2023 MacDougall W et al. *JAMA Network Open.*

eMethods. Codes used for all analyses described in the analytical section

```
# Read datasets

atv_inj_dad1 <-

  read_csv(here("data", "final_dad_icd10_2001-2014 combined.csv"), guess_max = 50000) %>%

  rename_with(to_snake_case) %>%

  rename(province = submitting_prov_code, discharge_year = fiscal_year,

         gender = gender_code, disposition = discharge_disposition) %>%

  mutate(age_group = if_else(age_units < 16, "<16",

         if_else(age_units>=21, "21+", "16-20"))) %>%

  mutate(urban_rural = case_when(substr(patient_postal_code_truncated, 2, 2) == "0" ~

        "RURAL/REMOTE", substr(patient_postal_code_truncated, 2, 2) %in% as.character(1:9) ~

        "URBAN")) %>%

  relocate(urban_rural, .after = patient_postal_code_truncated) %>%

  relocate(age_group,.after=age_units) %>%

  mutate(admission_year = substring(admission_date, 1, 4)) %>%

  mutate(admission_month=substring(admission_date, 6, 7))%>%

  relocate(admission_year, .after = admission_date)%>%

  relocate(admission_month, .after = admission_year)


dad_1 <- atv_inj_dad1 %>%

  select(study_id, province, age_group, gender, urban_rural,

         admission_year, admission_month, disposition, diag_code_1, diag_code_2, diag_code_3,

         diag_code_4, diag_code_5, diag_code_6, diag_code_7, diag_code_8, diag_code_9,
```

© 2023 MacDougall W et al. *JAMA Network Open.*

```
        diag_code_10,  total_los_days, acute_los_days, alc_los_days) %>%
mutate(disposition = as.character(disposition))



atv_inj_dad2 <-
  read_csv(here("data", "ATV_CIHI_2001_2019_no_pw.csv"), guess_max = 50000) %>%
  rename_with(to_snake_case) %>%
  rename(province = submitting_prov_code, discharge_year = fiscal_year,
        urban_rural = urban_rural_flag, age_group = age_grp, gender = gender_code,
        disposition = discharge_disposition) %>%
  mutate(admission_month_year = str_c("01", adm_month_year) %>% dmy()) %>%
  relocate(admission_month_year, .after = adm_month_year) %>%
  mutate(admission_year = substring(admission_month_year, 1, 4)) %>%
  mutate(admission_month=substring(admission_month_year, 6, 7))%>%
  relocate(admission_year, .after = admission_month_year)%>%
  relocate(admission_month, .after = admission_year)

dad_2 <- atv_inj_dad2 %>%
  select(study_id, province, age_group, gender, urban_rural, admission_year, admission_month,
      disposition, diag_code_1, diag_code_2, diag_code_3, diag_code_4, diag_code_5,
     diag_code_6, diag_code_7, diag_code_8, diag_code_9, diag_code_10,
     total_los_days, acute_los_days, alc_los_days)
```

© 2023 MacDougall W et al. *JAMA Network Open.*

Merge datasets

```
rowAny <- function(x) rowSums(x) > 0


merge <- bind_rows(dad_1, dad_2)


merge10 <- merge%>%
  pivot_longer(cols = contains("diag_code_"), names_to = "diag_code_field",
              values_to ="diag_code_value", values_drop_na = TRUE) %>%
  mutate(evaluable=case_when((admission_year == "1999")~0,
              (diag_code_value %>% str_detect("^V867"))~ 0, TRUE~1)) %>%


  mutate(missing=case_when((rowAny(across(.cols = everything(),.fns = ~ is.na(.x)))~0),
             TRUE~1))
```

Summarize Counts of Injuries

```
temp <- merge10%>%
  filter(evaluable=="1")%>%
  mutate(head_injury = diag_code_value %>% str_detect("^S06[0-9]")|
         diag_code_value %>% str_detect("^85[0-4]")) %>%
  mutate(fracture = diag_code_value %>% str_detect("^S[4-9]2[0-9]") |
         diag_code_value %>% str_detect("^T02[2-7]") |
         diag_code_value %>% str_detect("^T1[0,2]")|
```

© 2023 MacDougall W et al. *JAMA Network Open.*

```
        diag_code_value %>% str_detect("^8[1,2][0-9]")) %>%
  mutate(sci = diag_code_value %>% str_detect("^S[1-3]4[0,1]") |
        diag_code_value %>% str_detect("^S343")|
        diag_code_value %>% str_detect("^806")|
        diag_code_value %>% str_detect("^952")) %>%
  mutate(crush = diag_code_value %>% str_detect("^S[0,1,5]7[0-9]") |
        diag_code_value %>% str_detect("^S280") |
        diag_code_value %>% str_detect("^S[4,6,8,9]7[0-8]") |
        diag_code_value %>% str_detect("^S38[0,1]") |
        diag_code_value %>% str_detect("^S77[0,1,2]") |
        diag_code_value %>% str_detect("^T04[0-9]")|
        diag_code_value %>% str_detect("^92[5-9]")) %>%
  mutate(snowmobile =
        diag_code_value %in% c("U99045", "V8600", "V8650", "V8660", "V8690"),
       alcohol = diag_code_value %>% str_detect("^Y9"),
       driver = diag_code_value %in%
        c("V8600", "V8608", "V8651", "V8658"),
       passenger = diag_code_value %in%
        c("V8610", "V8618", "V8630", "V8638", "V8660", "V8668", "V8698"))

injuries <- temp %>%
  group_by(study_id, province, age_group, gender, urban_rural,
        admission_year, disposition, evaluable) %>%
```

© 2023 MacDougall W et al. *JAMA Network Open.*

```
  summarize(sci = any(sci), head_injury = any(head_injury), fracture = any(fracture),
            crush = any(crush), alcohol = any(alcohol), snowmobile = any(snowmobile),
            driver = any(driver), passenger = any(passenger))

injuries$driving_status <- with(injuries, ifelse(driver== TRUE, "driver", ifelse(passenger == TRUE,
                                         "passenger", "not reported")))
injuries$mortality <- with(injuries, ifelse((disposition == "7" | disposition == "72"), 1, 0))

# Descriptive Analysis
descriptive_dad <- injuries%>%
  mutate(admission_year=as.numeric(admission_year))%>%
  filter(admission_year %in% c(2002:2019))%>%
  mutate(year_group = case_when((admission_year>= 2002 & admission_year<=2006)~
             "2002-2006", (admission_year>= 2007 & admission_year<=2011)~ "2007-2011",
             (admission_year>= 2012 & admission_year<=2016)~ "2012-2016",
             (admission_year>= 2017 & admission_year<=2019)~ "2017-2019"))%>%
  mutate(province_group = case_when(province %in% c("0", "1", "2", "3")~ "NB/NS/PIE/NL",
              province == "5" ~ "ON", province %in% c("6", "7", "8", "9") ~ "BC/AB/SK/MB",
              province=="A"~ "territories"))

table(descriptive_dad$age_group)
table(descriptive_dad$gender, descriptive_dad$age_group)
table(descriptive_dad$province_group, descriptive_dad$age_group)
```

© 2023 MacDougall W et al. *JAMA Network Open.*

```
table(descriptive_dad$snowmobile, descriptive_dad$age_group)

table(descriptive_dad$urban_rural, descriptive_dad$age_group)


table(descriptive_dad$fracture, descriptive_dad$age_group)

table(descriptive_dad$head_injury, descriptive_dad$age_group)

table(descriptive_dad$crush, descriptive_dad$age_group)

table(descriptive_dad$sci, descriptive_dad$age_group)

table(descriptive_dad$mortality, descriptive_dad$age_group)


working_dad <- descriptive_dad%>% filter(province != "A")%>%

                    mutate(provine = as.numeric(province))


# ISS data prepared using SAS and merged with previous working data

load("R:/Active/McGavock - ATV Injury/data/ISS.Rdata")

merge_ISS_up <- merge_ISS_up%>%

          mutate(ISS = case_when((SASISS_10diag>=0 & SASISS_10diag<=15)~1,

                (SASISS_10diag>15 & SASISS_10diag<=25)~2,

                (SASISS_10diag>25)~3))


merge_ISS_up$ISS2 <- ifelse(merge_ISS_up$ISS==3, 1, 0)


table(merge_ISS_up$ISS2, merge_ISS_up$age_group)
```

© 2023 MacDougall W et al. *JAMA Network Open.*

```
# Model Fit

# Model Fit M1 (only age, null model)

head_fit_M1 <- glm(head_injury~ age_group, data = working_dad, family = "binomial")

sci_fit_M1 <- glm(sci~ age_group, data = working_dad, family = "binomial")

crush_fit_M1 <- glm(crush~ age_group, data = working_dad, family = "binomial")


fracture_fit_M1 <- glm(fracture~ age_group, data = working_dad, family = "binomial")

mortality_fit_M1 <- glm(mortality~ age_group , data = working_dad, family = "binomial")

ISS_fit_M1 <- glm(ISS2~age_group, data = merge_ISS_up, family = "binomial")


head_M1 <- head_fit_M1%>%tidy(exp=TRUE, conf.int=TRUE)%>%

                    filter(term%>%str_detect("age"))

sci_M1 <- sci_fit_M1%>%tidy(exp=TRUE, conf.int=TRUE)%>%filter(term%>%str_detect("age"))

crush_M1 <- crush_fit_M1%>%tidy(exp=TRUE, conf.int=TRUE)%>%

                    filter(term%>%str_detect("age"))

fracture_M1 <- fracture_fit_M1%>%tidy(exp=TRUE, conf.int=TRUE)%>%

                    filter(term%>%str_detect("age"))

mortality_M1 <- mortality_fit_M1%>%tidy(exp=TRUE, conf.int=TRUE)%>%

                    filter(term%>%str_detect("age"))

ISS_M1 <- ISS_fit_M1%>%tidy(exp=TRUE, conf.int=TRUE)%>%filter(term%>%str_detect("age"))


inj_type <- c(rep("Head", 2), rep("SCI", 2), rep("Crush", 2), rep("Fracture", 2), rep("Death", 2),

                    rep("ISS>25", 2))
```

© 2023 MacDougall W et al. *JAMA Network Open.*

```
M1_table <- cbind(inj_type, rbind(head_M1, sci_M1, crush_M1, fracture_M1, mortality_M1,
                    ISS_M1))
```

Model Fit M2 (age + covariates + snowmobile + driving status)

```
head_fit_M2 <- glm(head_injury~ year_group + province_group  + age_group + gender +
                    urban_rural + snowmobile + driver, data = working_dad, family = "binomial")
sci_fit_M2 <- glm(sci~ year_group + province_group  + age_group + gender + urban_rural+
                    snowmobile + driver, data = working_dad, family = "binomial")
crush_fit_M2 <- glm(crush~ year_group + province_group  + age_group + gender +
                    urban_rural+ snowmobile + driver, data = working_dad, family = "binomial")

fracture_fit_M2 <- glm(fracture~ year_group + province_group  + age_group + gender +
                    urban_rural+ snowmobile + driver, data = working_dad, family = "binomial")
mortality_fit_M2 <- glm(mortality~ year_group + province_group  + age_group + gender +
                    urban_rural+ snowmobile + driver, data = working_dad, family = "binomial")
ISS_fit_M2 <- glm(ISS2~year_group + province_group  + age_group + gender + urban_rural+
                    snowmobile + driver, data = merge_ISS_up, family = "binomial")

head_M2 <- head_fit_M2%>%tidy(exp=TRUE, conf.int=TRUE)%>%
                    filter(term%>%str_detect("age"))
sci_M2 <- sci_fit_M2%>%tidy(exp=TRUE, conf.int=TRUE)%>%filter(term%>%str_detect("age"))
crush_M2 <- crush_fit_M2%>%tidy(exp=TRUE, conf.int=TRUE)%>%
```

© 2023 MacDougall W et al. *JAMA Network Open.*

```
            filter(term%>%str_detect("age"))
fracture_M2 <- fracture_fit_M2%>%tidy(exp=TRUE, conf.int=TRUE)%>%
            filter(term%>%str_detect("age"))
mortality_M2 <- mortality_fit_M2%>%tidy(exp=TRUE, conf.int=TRUE)%>%
            filter(term%>%str_detect("age"))
ISS_M2 <- ISS_fit_M2%>%tidy(exp=TRUE, conf.int=TRUE)%>%filter(term%>%str_detect("age"))

M2_table <- cbind(inj_type,rbind(head_M2, sci_M2, crush_M2, fracture_M2, mortality_M2,
            ISS_M2))

# Model Fit M3 (M2 + gender*age)
head_fit_M3 <- glm(head_injury~ year_group + province_group  + age_group + gender +
            urban_rural+ snowmobile + driver + gender*age_group, data = working_dad, family =
            "binomial")
sci_fit_M3 <- glm(sci~ year_group + province_group  + age_group + gender + urban_rural+
            snowmobile + driver + gender*age_group, data = working_dad, family =
            "binomial")

crush_fit_M3 <- glm(crush~ year_group + province_group  + age_group + gender +
             urban_rural+ snowmobile + driver + gender*age_group, data = working_dad,
             family = "binomial")
fracture_fit_M3 <- glm(fracture~ year_group + province_group  + age_group + gender +
             urban_rural+ snowmobile + driver + gender*age_group, data = working_dad,
```

© 2023 MacDougall W et al. *JAMA Network Open.*

```
                         family = "binomial")
mortality_fit_M3 <- glm(mortality~ year_group + province_group  + age_group + gender +
                          urban_rural+ snowmobile + driver + gender*age_group, data = working_dad,
                          family = "binomial")
ISS_fit_M3 <- glm(ISS2~year_group + province_group  + age_group + gender + urban_rural+
                         snowmobile + driver + gender*age_group, data = merge_ISS_up, family =
                         "binomial")

head_M3 <- head_fit_M3%>%tidy(exp=TRUE, conf.int=TRUE)%>%
                     filter(term%>%str_detect("age"))
sci_M3 <- sci_fit_M3%>%tidy(exp=TRUE, conf.int=TRUE)%>%filter(term%>%str_detect("age"))
crush_M3 <- crush_fit_M3%>%tidy(exp=TRUE, conf.int=TRUE)%>%
                     filter(term%>%str_detect("age"))
fracture_M3 <- fracture_fit_M3%>%tidy(exp=TRUE, conf.int=TRUE)%>%
                     filter(term%>%str_detect("age"))
mortality_M3 <- mortality_fit_M3%>%tidy(exp=TRUE, conf.int=TRUE)%>%
                     filter(term%>%str_detect("age"))
ISS_M3 <- ISS_fit_M3%>%tidy(exp=TRUE, conf.int=TRUE)%>%filter(term%>%str_detect("age"))

inj_type2 <- c(rep("Head", 4), rep("SCI", 4), rep("Crush", 4), rep("Fracture", 4), rep("Death", 4),
                     rep("ISS>25", 4))

M3_table <- cbind(inj_type2, gender = rep(c("Females", "Females", "Males", "Males"), 6),
```

© 2023 MacDougall W et al. *JAMA Network Open.*

```
                    age_grp =rep(c("16-20", "21+"), 12), rbind(head_M3, sci_M3, crush_M3,
                    fracture_M3, mortality_M3, ISS_M3))
M3_table$inj_type2<-factor(M3_table$inj_type2, levels = c("Fracture", "Crush",  "Head",
                    "Death", "SCI", "ISS>25"  ))
```

Model Fit M4 (M2 + age*snowmobile)

```
head_fit_M4 <- glm(head_injury~ year_group + province_group  + age_group + gender +
                    urban_rural+ snowmobile + driver + snowmobile*age_group, data =
                    working_dad, family = "binomial")
sci_fit_M4 <- glm(sci~ year_group + province_group  + age_group + gender + urban_rural+
                    snowmobile + driver + snowmobile*age_group, data = working_dad, family =
                    "binomial")
crush_fit_M4 <- glm(crush~ year_group + province_group  + age_group + gender +
                      urban_rural+ snowmobile + driver + snowmobile*age_group, data = working_dad,
                    family = "binomial")
fracture_fit_M4 <- glm(fracture~ year_group + province_group  + age_group + gender +
                    urban_rural+ snowmobile + driver + snowmobile*age_group, data = working_dad,
                    family = "binomial")
mortality_fit_M4 <- glm(mortality~ year_group + province_group  + age_group + gender +
                    urban_rural+ snowmobile + driver + snowmobile*age_group, data = working_dad,
                    family = "binomial")
ISS_fit_M4 <- glm(ISS2~year_group + province_group  + age_group + gender + urban_rural+
                    snowmobile + driver + snowmobile*age_group, data = merge_ISS_up, family =
                    "binomial")
```

© 2023 MacDougall W et al. *JAMA Network Open.*

```
head_M4 <- head_fit_M4%>%tidy(exp=TRUE, conf.int=TRUE)%>%
                        filter(term%>%str_detect("age"))
sci_M4 <- sci_fit_M4%>%tidy(exp=TRUE, conf.int=TRUE)%>%filter(term%>%str_detect("age"))
crush_M4 <- crush_fit_M4%>%tidy(exp=TRUE, conf.int=TRUE)%>%
                        filter(term%>%str_detect("age"))
fracture_M4 <- fracture_fit_M4%>%tidy(exp=TRUE, conf.int=TRUE)%>%
                        filter(term%>%str_detect("age"))
mortality_M4 <- mortality_fit_M4%>%tidy(exp=TRUE, conf.int=TRUE)%>%
                        filter(term%>%str_detect("age"))
ISS_M4 <- ISS_fit_M4%>%tidy(exp=TRUE, conf.int=TRUE)%>%filter(term%>%str_detect("age"))

M4_table <- cbind(inj_type2, snowmobile = rep(c("Snowmobile: Not Reported",
                        "Snowmobile: Not Reported", "Snowmobile: Yes", "Snowmobile: Yes"), 6), age_grp
                        =rep(c("16-20", "21+"), 12), rbind(head_M4, sci_M4, crush_M4, fracture_M4,
                        mortality_M4, ISS_M4))
M4_table$inj_type2<-factor(M4_table$inj_type2, levels = c("Fracture", "Crush",  "Head",
                        "Death", "SCI", "ISS>25"  ))

# Model Fit M4 (M2 + driving*age)
head_fit_M5 <- glm(head_injury~  year_group + province_group  + age_group + gender +
                                urban_rural+ snowmobile + driver + driver*age_group, data = working_dad,
                                family = "binomial")
sci_fit_M5<- glm(sci~  year_group + province_group  + age_group + gender + urban_rural+
```

© 2023 MacDougall W et al. *JAMA Network Open.*

```
                     snowmobile + driver + driver*age_group, data = working_dad, family =
                     "binomial")
crush_fit_M5 <- glm(crush~ year_group + province_group  + age_group + gender +
                     urban_rural+ snowmobile + driver + driver*age_group, data = working_dad,
                     family = "binomial")
fracture_fit_M5 <- glm(fracture~ year_group + province_group  + age_group + gender +
                     urban_rural+ snowmobile + driver + driver*age_group, data = working_dad,
                     family = "binomial")


mortality_fit_M5 <- glm(mortality~ year_group + province_group  + age_group + gender +
                     urban_rural+ snowmobile + driver + driver*age_group, data = working_dad,
                     family = "binomial")
ISS_fit_M5 <- glm(ISS2~year_group + province_group  + age_group + gender + urban_rural+
                     snowmobile + driver + driver*age_group, data = merge_ISS_up, family =
                     "binomial")


head_M5 <- head_fit_M5%>%tidy(exp=TRUE, conf.int=TRUE)%>%
                     filter(term%>%str_detect("age"))
sci_M5 <- sci_fit_M5%>%tidy(exp=TRUE, conf.int=TRUE)%>%filter(term%>%str_detect("age"))
crush_M5 <- crush_fit_M5%>%tidy(exp=TRUE, conf.int=TRUE)%>%
                     filter(term%>%str_detect("age"))
fracture_M5 <- fracture_fit_M5%>%tidy(exp=TRUE, conf.int=TRUE)%>%
                     filter(term%>%str_detect("age"))
mortality_M5 <- mortality_fit_M5%>%tidy(exp=TRUE, conf.int=TRUE)%>%
```

© 2023 MacDougall W et al. *JAMA Network Open.*

```
                    filter(term%>%str_detect("age"))
ISS_M5 <- ISS_fit_M5%>%tidy(exp=TRUE, conf.int=TRUE)%>%filter(term%>%str_detect("age"))


M5_table <- cbind(inj_type2, driving_status = rep(c("Passenger/Not Reported",
          "Passenger/Not Reported", "Driver", "Driver"), 6),
          age_grp =rep(c("16-20", "21+"), 12), rbind(head_M5, sci_M5, crush_M5, fracture_M5,
          mortality_M5, ISS_M5))
M5_table$inj_type2<-factor(M5_table$inj_type2, levels = c("Fracture", "Crush",  "Head",
          "Death", "SCI", "ISS>25"  ))
```

```
# Figures:
dg = position_dodge(width = 0.5)
M1_table$inj_type<-factor(M1_table$inj_type, levels = c("Fracture", "Crush",  "Head",
          "Death", "SCI", "ISS>25"  ))
fig1 <-ggplot(M1_table, aes(x=inj_type, y=estimate))+
          geom_errorbar(aes(ymin=conf.low, ymax=conf.high, linetype = term),
          width=.1, position = dg, cex = 1) +
          geom_point(aes(shape=term), position=dg, size = 4)+
          coord_flip(ylim=c(0.3,10)) + geom_hline(yintercept=1, cex = 1)+
```

© 2023 MacDougall W et al. *JAMA Network Open.*

```
                        ylab("Odds Ratio (95% CI)") + xlab("Injury Types") +
                        scale_y_log10() +
                        theme(panel.background = element_rect(fill = 'gray95', colour = 'black'),
                        axis.text=element_text(size=14,face="bold"),
                        axis.title=element_text(size=16,face="bold"),
                        axis.text.x = element_text(angle = 45),
                      axis.line = element_line(colour = "black", size = 1),
                       legend.text = element_text(size=14),
                        legend.title = element_text(size=14),
                        legend.position = "bottom")+
                        guides(size = "none")+
                        scale_shape_discrete(name  ="Age Groups:",
                       labels=c("16-20 vs. <16", "21+ vs. <16"))+
                        scale_linetype_discrete(name  ="Age Groups:",
                       labels=c("16-20 vs. <16", "21+ vs. <16"))

M2_table$inj_type<-factor(M2_table$inj_type, levels = c("Fracture", "Crush",  "Head",
                        "Death", "SCI", "ISS>25"  ))



fig2 <-ggplot(M2_table, aes(x=inj_type, y=estimate))+
                        geom_errorbar(aes(ymin=conf.low, ymax=conf.high, linetype = term),
                        width=.1, position = dg, cex = 1) +
```

© 2023 MacDougall W et al. *JAMA Network Open.*

```
                  geom_point(aes(shape=term), position=dg, size = 4)+
                  coord_flip(ylim=c(0.3,10)) + geom_hline(yintercept=1, cex = 1)+
                  ylab("Odds Ratio (95% CI)") + xlab("Injury Types") +
                  scale_y_log10() +
                  theme(panel.background = element_rect(fill = 'gray95', colour = 'black'),
                  axis.text=element_text(size=14,face="bold"),
                  axis.title=element_text(size=16,face="bold"),
                  axis.text.x = element_text(angle = 45),
                 axis.line = element_line(colour = "black", size = 1),
                  legend.text = element_text(size=14), legend.title = element_text(size=14),
                  legend.position = "bottom") + guides(size = "none")+
                  scale_shape_discrete(name  ="Age Groups:",
                 labels=c("16-20 vs. <16", "21+ vs. <16"))+
                  scale_linetype_discrete(name  ="Age Groups:",
                 labels=c("16-20 vs. <16", "21+ vs. <16"))


fig3 <- ggplot(M3_table, aes(x=inj_type2, y=estimate)) +
                  geom_point(position = dg, aes(shape=age_grp), size = 4)+
                   geom_errorbar(aes(ymin=conf.low, ymax=conf.high, linetype = age_grp),
                  width=0.1, position = dg, cex = 1) +
                  facet_grid(~gender)+ coord_flip() + geom_hline(yintercept=1, cex = 1)+
                  scale_y_log10() + ylab("Odds Ratio (95% CI)") + xlab("Injury Types") +
                  theme(panel.background = element_rect(fill = 'gray95', colour = 'black'),
```

© 2023 MacDougall W et al. *JAMA Network Open.*

```
                    axis.text=element_text(size=14,face="bold"),
                    axis.title=element_text(size=16,face="bold"),
                    axis.line = element_line(colour = "black", size = 1),
                    legend.text = element_text(size=14), legend.title = element_text(size=14),
                    legend.position = "bottom", strip.text = element_text(size = 14, face="bold"))+
                    theme(strip.background =element_rect(fill="white"))+guides(size = "none")+
                    scale_shape_discrete(name  ="Age Groups:",
                   labels=c("16-20 vs. <16", "21+ vs. <16"))+
                    scale_linetype_discrete(name  ="Age Groups:",
                   labels=c("16-20 vs. <16", "21+ vs. <16"))

M4_table$snowmobile<-factor(M4_table$snowmobile, levels = c("Snowmobile: Yes",
                    "Snowmobile: Not Reported" ))
fig4 <- ggplot(M4_table, aes(x=inj_type2, y=estimate)) +
                    geom_point(position = dg, aes(shape=age_grp), size = 4)+
                    geom_errorbar(aes(ymin=conf.low, ymax=conf.high, linetype = age_grp),
          width=0.1, position = dg, cex = 1) +
                    facet_grid(~snowmobile)+ coord_flip() + geom_hline(yintercept=1, cex = 1)+
                    scale_y_log10() +  ylab("Odds Ratio (95% CI)") + xlab("Injury Types") +
                    theme(panel.background = element_rect(fill = 'gray95', colour = 'black'),
                    axis.text=element_text(size=14,face="bold"),
                    axis.title=element_text(size=16,face="bold"),
                    axis.line = element_line(colour = "black", size = 1),
```

© 2023 MacDougall W et al. *JAMA Network Open.*

```
                    legend.text = element_text(size=14),
                    legend.title = element_text(size=14),
                    legend.position = "bottom",
                    strip.text = element_text(size = 14, face="bold"))+
                    theme(strip.background =element_rect(fill="white"))+
                    guides(size = "none")+ scale_shape_discrete(name  ="Age Groups:",
                    labels=c("16-20 vs. <16", "21+ vs. <16"))+
                    scale_linetype_discrete(name  ="Age Groups:",
                  labels=c("16-20 vs. <16", "21+ vs. <16"))
M5_table$driving_status<-factor(M5_table$driving_status, levels = c("Driver",
                         "Passenger/Not Reported" ))
fig5 <- ggplot(M5_table, aes(x=inj_type2, y=estimate)) +
                    geom_point(position = dg, aes(shape=age_grp), size = 4)+
                    geom_errorbar(aes(ymin=conf.low, ymax=conf.high, linetype = age_grp),
                    width=0.1, position = dg, cex = 1) +
                    facet_grid(~driving_status)+
                    coord_flip() + geom_hline(yintercept=1, cex = 1)+ scale_y_log10() +
                    ylab("Odds Ratio (95% CI)") + xlab("Injury Types") +
                    theme(panel.background = element_rect(fill = 'gray95', colour = 'black'),
                    axis.text=element_text(size=14,face="bold"),
                    axis.title=element_text(size=16,face="bold"),
                    axis.line = element_line(colour = "black", size = 1),
                    legend.text = element_text(size=14),
```

© 2023 MacDougall W et al. *JAMA Network Open.*

```
                legend.title = element_text(size=14), legend.position = "bottom",
                strip.text = element_text(size = 14, face="bold"))+
                theme(strip.background =element_rect(fill="white"))+guides(size = "none")+
                scale_shape_discrete(name  ="Age Groups:",
                labels=c("16-20 vs. <16", "21+ vs. <16"))+
                scale_linetype_discrete(name  ="Age Groups:",
               labels=c("16-20 vs. <16", "21+ vs. <16"))


# Test Significance of interactions
Gen_int_test = rbind(as.data.frame(anova(head_fit_M3, test = "Chisq"))[9,],
   as.data.frame(anova(sci_fit_M3, test = "Chisq"))[9,],
   as.data.frame(anova(fracture_fit_M3, test = "Chisq"))[9,],
   as.data.frame(anova(crush_fit_M3, test = "Chisq"))[9,],
   as.data.frame(anova(mortality_fit_M3, test = "Chisq"))[9,],
   as.data.frame(anova(ISS_fit_M3, test = "Chisq"))[9,])


data.frame(Injury = c("Head", "SCI", "Fracture", "Crush", "Death", "ISS"), Gen_int_test)


Snow_int_test = rbind(as.data.frame(anova(head_fit_M4, test = "Chisq"))[9,],
   as.data.frame(anova(sci_fit_M4, test = "Chisq"))[9,],
   as.data.frame(anova(fracture_fit_M4, test = "Chisq"))[9,],
   as.data.frame(anova(crush_fit_M4, test = "Chisq"))[9,],
   as.data.frame(anova(mortality_fit_M4, test = "Chisq"))[9,],
```

© 2023 MacDougall W et al. *JAMA Network Open.*

```
    as.data.frame(anova(ISS_fit_M4, test = "Chisq"))[9,])

data.frame(Injury = c("Head", "SCI", "Fracture", "Crush", "Death", "ISS"), Snow_int_test)

Driv_int_test = rbind(as.data.frame(anova(head_fit_M5, test = "Chisq"))[9,],
    as.data.frame(anova(sci_fit_M5, test = "Chisq"))[9,],
    as.data.frame(anova(fracture_fit_M5, test = "Chisq"))[9,],
    as.data.frame(anova(crush_fit_M5, test = "Chisq"))[9,],
    as.data.frame(anova(mortality_fit_M5, test = "Chisq"))[9,],
    as.data.frame(anova(ISS_fit_M5, test = "Chisq"))[9,])

data.frame(Injury = c("Head", "SCI", "Fracture", "Crush", "Death", "ISS"), Driv_int_test)
```

SAS Codes

```
%let projectdir = R:\Active\McGavock - ATV Injury;

libname ref "&projectdir.\SAS - ISS calcs";
libname data "&projectdir.\data";
* filename: cross_walk_icd10_iss_for clients_overalISS_2021-08-17.sas;
* last mod: 2021-08-17 (SS);


*********************************************************************
PURPOSE: CrossWalking Avery_98_Low to compute ISS using ICD10 codes

- use this file to generate ISS for dad dataset*
```

© 2023 MacDougall W et al. *JAMA Network Open.*

```
**********************************************************************;
* create SASISS using 10 diagnoses (2021-08 analyses);
%let n_diag = 10;


*****************************************
* Xwalk starts with dataset "PAT_Xwalk" *
*****************************************;
* Step 1: identify ICD10 codes;

* load our data:
      import dad data(2001-2020) and renamed as PAT_Xwalk
      note: the data was combined by 2001-15 and 1999-2020 in R;

PROC IMPORT OUT= WORK.PAT_XWALK
            DATAFILE= "R:\Active\McGavock - ATV
Injury\data\merge_dad_data_transpose.csv"
            DBMS=CSV REPLACE;
     GETNAMES=YES;
     DATAROW=2;
     GUESSINGROWS=230000;
RUN;

*create the SASISS using only first n_diag diagnoses;
*data PAT_Xwalk_relevant_diagnoses;
*     set PAT_Xwalk;
      *%if &n_diag. ne 25 %then %do;
            *drop DIAG_CODE_&n1_diag.-DIAG_CODE_25;
      *%end;
*run;

/*comment this part out when needs to use all 25 diagnoses*/
```

© 2023 MacDougall W et al. *JAMA Network Open.*

```
*data PAT_WALK;
*     set PAT_Xwalk_relevant_diagnoses;
*run;



*prepare for transpose;
*proc sort data=PAT_Xwalk;
*     by study_id;
      *where diag_code_1 ne "";
*run;

*check any dup in ID;
proc sort data=PAT_Xwalk out=nodup dupout=dup nodupkey;
      by study_id;
 run;*no dups in NTR dataset;

* I transposed in R and checked duplicate or missing diag_code_1 case. There
was not any.

/*comment this part out when needs to use all 25 diagnoses*/
*PROC TRANSPOSE DATA=PAT_Xwalk OUT=PAT_Xwalk_1;
    * BY    study_id ; * the variable for patient ID;
      * VAR diag_code_1-diag_code_&n_diag.;
       * you might need to modify this VAR statement, depending how many
ICD10 for patients,
       and the name of ICD10 in your file;
*RUN;*301075;




proc freq data=PAT_Xwalk;
```

© 2023 MacDougall W et al. *JAMA Network Open.*

```
table diag_code_value;
run;

* keep only trauma injury with S or T codes;
DATA PAT_Xwalk_1;
    SET PAT_Xwalk;
      ICD_10_Code=diag_code_value;
      IF SUBSTR(ICD_10_Code,1,1) IN ('S', 'T');
      DROP diag_code_field;
      RUN;*118094;

*********
* Step 2: sort ICD_10_code codes for merging in Step 3;
proc sort data=PAT_XWALK_1;
by ICD_10_code;
run;*118094;

*********
* Step 3: CrossWalk ICD_10_code codes to get Ais Score at each ISS Region;

* check any dups in ref.Icd10_ais_updated2009;
proc sql;
      create table temp as
      select ICD_10_code, count (*) as n_dup
      from ref.Icd10_ais_updated2009
      group by ICD_10_code
      having n_dup>1;

      create table ref_dups_1 as
      select * from ref.Icd10_ais_updated2009
      where ICD_10_code in (select ICD_10_code from temp);  *s064, s065, s069
have duplicate record;
```

© 2023 MacDougall W et al. *JAMA Network Open.*

```
quit; *they are exact copy of themselves => remove 3 records;


*remove dups;

proc sort data=ref.Icd10_ais_updated2009 out=Icd10_ais_updated2009 nodupkey;
*1572;

      by ICD_10_code _ALL_;

run;*1569;


*the following step is equivalent to inner join;

data Cross_walk;

merge PAT_XWALK_2 Icd10_ais_updated2009;

by ICD_10_code;

if study_id  NE ""; * here study_id  is the patient ID ;

if ISS_body_region NE '.';

if AIS_98_Low NE '.';

keep study_id  ICD_10_code ISS_Body_region AIS_98_Low;

run;*21336 < 23494 (2158 less records);


* find those 2158 loss records;

proc freq data=PAT_XWALK_2 noprint;

      table ICD_10_Code/out=b_2; *1199;

run;


proc freq data=Cross_walk noprint;

      table ICD_10_Code/out=a_2; *985;

run;

*1199-985=214 not matched, missings not found;


* Step 4: Sort by Patient ID, ISS region and Ais score;

proc sort data=Cross_walk out=Cross_walk_1;

by study_id ISS_Body_region AIS_98_Low;

run;*21336;
```

© 2023 MacDougall W et al. *JAMA Network Open.*

```
***********************************************************************
* Step 5: Max-AIS by ISS region with adjustment for pelvic fractures;
***********************************************************************;
* A;
data Cross_walk_2A;
set Cross_walk_1;

if ICD_10_Code in ('S32500', 'S334') then Count_1=1; * use 'IN' for "OR"
statement;
if ICD_10_Code in ('S332', 'S32100') then Count_2=1;

if ICD_10_Code in ('S32500', 'S334') then Count_3=1;
if ICD_10_Code in ('S32101') then Count_4=1;

if ICD_10_Code in ('S32501') then Count_5=1;
if ICD_10_Code in ('S332', 'S32100') then Count_6=1;

if ICD_10_Code in ('S32501') then Count_7=1;
if ICD_10_Code in ('S32101') then Count_8=1;
run;*21336;

* B;
data Cross_walk_2B;
set Cross_walk_2A;

Sum_Count_1+Count_1; * new variable sum up the counts for each patient;
Sum_Count_2+Count_2;
Sum_Count_3+Count_3;
Sum_Count_4+Count_4;
Sum_Count_5+Count_5;
```

© 2023 MacDougall W et al. *JAMA Network Open.*

```
Sum_Count_6+Count_6;

Sum_Count_7+Count_7;

Sum_Count_8+Count_8;


by study_id;


if FIRST.study_id then Sum_Count_1=Count_1; * reset the Sum to the starting
value for each patient;

if FIRST.study_id then Sum_Count_2=Count_2;

if FIRST.study_id then Sum_Count_3=Count_3;

if FIRST.study_id then Sum_Count_4=Count_4;

if FIRST.study_id then Sum_Count_5=Count_5;

if FIRST.study_id then Sum_Count_6=Count_6;

if FIRST.study_id then Sum_Count_7=Count_7;

if FIRST.study_id then Sum_Count_8=Count_8;



if Sum_Count_1>=1 AND Sum_Count_2>=1 then AIS_Pelvic_Low=4;  * 'AND'
statement;

if Sum_Count_1>=1 AND Sum_Count_2>=1 then AIS_Pelvic_Medium=4;

if Sum_Count_1>=1 AND Sum_Count_2>=1 then AIS_Pelvic_High=5;


* note the order of the coding paragraphs above and beLow,

"AIS_Pelvic_Low=5" should be after "AIS_Pelvic_Low=4",

ensure that "AIS_Pelvic_Low=5" NOT be replaced;


if Sum_Count_3>=1 AND Sum_Count_4>=1 then AIS_Pelvic_Low=5;  * 'AND'
statement;

if Sum_Count_3>=1 AND Sum_Count_4>=1 then AIS_Pelvic_Medium=5;

if Sum_Count_3>=1 AND Sum_Count_4>=1 then AIS_Pelvic_High=5;


if Sum_Count_5>=1 AND Sum_Count_6>=1 then AIS_Pelvic_Low=5;  * 'AND'
statement;

if Sum_Count_5>=1 AND Sum_Count_6>=1 then AIS_Pelvic_Medium=5;
```

© 2023 MacDougall W et al. *JAMA Network Open.*

```
if Sum_Count_5>=1 AND Sum_Count_6>=1 then AIS_Pelvic_High=5;

if Sum_Count_7>=1 AND Sum_Count_8>=1 then AIS_Pelvic_Low=5;  * 'AND'
statement;
if Sum_Count_7>=1 AND Sum_Count_8>=1 then AIS_Pelvic_Medium=5;
if Sum_Count_7>=1 AND Sum_Count_8>=1 then AIS_Pelvic_High=5;

run;*21336;

* C;
* keep Max_AIS (LAST.) at each ISS region *;
data Cross_walk_2C;
set Cross_walk_2B;
X+1;
by study_id ISS_Body_Region;
if LAST.ISS_Body_Region;
drop X ICD_10_Code count_1-count_8 sum_count_1-sum_count_8;
run; *15065;

* D: adjustment for pelvic fracture;
* max AIS by ICD10 mapping;
data Cross_walk_2D;
set Cross_walk_2C;

if 6>=ISS_Body_Region>=1; * drop un-specified region 9 *;

* this statment adjusts pelvic fracture;
* pelvis adjustment occurs only at ISS region 6;
if ISS_Body_Region=5 AND AIS_Pelvic_Medium NE '.' then AIS_98_Low =
AIS_Pelvic_Low;

keep study_id ISS_Body_Region AIS_98_Low;
run; *15065;
```

© 2023 MacDougall W et al. *JAMA Network Open.*

```
*********
* Step 6: sort AIS_98_Low by descending order;
proc sort data=Cross_walk_2D out=Cross_walk_3;
by study_id  descending AIS_98_Low;
run; *15065;

*********
* Step 7: count how many ISS regions;
data Cross_walk_4;
set Cross_walk_3;
Count_region + 1 ;
by study_id  ;
if FIRST.study_id  then Count_region = 1;
run; *15065;

*********
* Step 8: keep only the three ISS regions that have most severest injuries;
data Cross_walk_5;
set Cross_walk_4;
if Count_region>=4 then delete;
run;*14843;

*********
* Step 9: compute ISS, the sum of square of Ais score for the three most
severest ISS regions;
data Cross_walk_6;
set Cross_walk_5;
AIS_square= AIS_98_Low*AIS_98_Low;
ISS + AIS_square; *adding all AIS_square for one person together in long
format;
by study_id  Count_Region;
```

© 2023 MacDougall W et al. *JAMA Network Open.*

```
if FIRST.study_id  then ISS = AIS_square;

if ISS>75 then ISS=75; * maximum ISS is 75;

if AIS_98_Low=6 then ISS=75; * an Ais=6 is automatically ISS=75;

run;*14843;


*********

* Step 10: keep the final ISS for each patient - only the last record with
the cummunative ISS;

data Cross_walk_7;

set Cross_walk_6;

Y+1; * used for BY statement below;

BY study_id  Count_Region;

if LAST.study_id ;

keep study_id ISS;

* NOW you have the ISS for each patient study_id;

run;*11576;


*rename the ISS;

data Cross_walk_&n_diag.diag;

      set Cross_walk_7;

      rename ISS = SASISS_&n_diag.diag;

run;


*here, both SASISS of 3 diagnoses and 25 diagnoses are wanted by clients,
therefore, merge the two

      datasets to have both SASISS scores;

proc sql;

      create table Cross_walk_8 as

      select * from CROSS_WALK_3DIAG A

      right join CROSS_WALK_25DIAG B

      on A.study_id = B.study_id;

quit;*11576;
```

© 2023 MacDougall W et al. *JAMA Network Open.*

```
*export the dataset;
proc export data=Cross_walk_8
      outfile="&projectdir.\data\DAD_2001_14_SASISS.csv"
      dbms=csv replace;
run;
```

© 2023 MacDougall W et al. *JAMA Network Open.*
