# Supplementary material for: Severity of All-Terrain Vehicle–Related Injuries by Age in Canada, 2002-2019
Source: JAMA Netw Open. 2023 May 31;6(5):e2316060. doi: 10.1001/jamanetworkopen.2023.16060 (PMC10233422; doi:10.1001/jamanetworkopen.2023.16060)
Supplement: Supplement 2. — Data Sharing Statement [file jamanetwopen-e2316060-s002.pdf]

Data Sharing Statement

MacDougall. Severity of All-Terrain Vehicle-Related Injuries by Age in Canada, 2002-2019. *JAMA Netw Open*. Published May 31, 2023. doi:10.1001/jamanetworkopen.2023.16060

Data
Data available: No

Additional Information
Explanation for why data not available: The data set from this study is held securely at the Canadian Institutes for Health Information (CIHI). Under a data-sharing agreement with CIHI we are prohibited from making the data set publicly available.
